# Identification and Characterization of a Large Effect QTL from *Oryza glumaepatula* Revealed *Pi68(t)* as Putative Candidate Gene for Rice Blast Resistance

**DOI:** 10.1186/s12284-020-00378-4

**Published:** 2020-03-12

**Authors:** S. J. S. Rama Devi, Kuldeep Singh, B. Umakanth, B. Vishalakshi, K. Vijaya Sudhakara Rao, B. Suneel, S. K. Sharma, Gopala Krishna Murthy Kadambari, M. S. Prasad, P. Senguttvel, Divya P. Syamaladevi, M. S. Madhav

**Affiliations:** 1 Crop Improvement Division, Indian Institute of Rice Research, Hyderabad-30, India; 2 Department of Plant Breeding and Genetics, P.A.U, Ludhiana, Punjab India; 3 ICAR-National Bureau of Plant Genetic Resources, New Delhi, India; 4 School of Agricultural Biotechnology, P.A.U, Ludhiana, Punjab India; 5 Plant Pathology Division, ICAR Research Complex for NEH Region, Manipur Centre, Imphal, India; 6 Plant Pathology Division, Indian Institute of Rice Research, Hyderabad-30, India; 7 Crop Improvement Section, IIRR, Hyderabad, 500 030 India

**Keywords:** Field resistance, Leaf and neck blast resistance, *Magnaporthe oryzae*, Marker-assisted backcrossing, *O. glumaepatula*, Quantitative trait loci

## Abstract

**Background:**

Field resistance is often effective and durable as compared to vertical resistance. The introgression line (INGR15002) derived from O. glumaepatula has proven broad spectrum field resistance for both leaf and neck blast.

**Results:**

Quantitative Trait Loci (QTL) analysis of INGR15002, led to the identification of two major QTL - qBL3 contributing about 34% and 32% phenotypic variance towards leaf and neck blast resistance, respectively and qBL7 contributing about 25% of phenotypic variance for leaf blast. Further, qBL3 was fine mapped, narrowed down to 300 kb region and a linked SNP maker was identified. By combining mapping with microarray analysis, a candidate gene, Os03g0281466 (malectin-serine threonine kinase), was identified in the fine mapped region and named as Pi68(t). The nucleotide variations in the coding as well as upstream region of the gene was identified through cloning and sequence analysis of Pi68(t) alleles. These significant variations led to the non-synonymous changes in the protein as well as variations (presence/absence) in four important motifs (W-box element; MYC element; TCP element; BIHD1OS) at promoter region those are associated with resistance and susceptible reactions. The effect of qBL3 was validated by its introgression into BPT5204 (susceptible variety) through marker-assisted selection and progeny exhibiting resistance to both leaf and neck blast was identified. Further, the utility of linked markers of Pi68(t) in the blast breeding programs was demonstrated in elite germplasm lines.

**Conclusions:**

This is the first report on the identification and characterization of major effect QTL from O. glumaepatula, which led to the identification of a putative candidate gene, Pi68(t), which confers field resistance to leaf as well as neck blast in rice.

## Background

Rice blast caused by *Magnaporthe oryzae*, is one of the most destructive diseases that affects the crop, right from the vegetative stage (leaf blast) to the reproductive stage (neck blast) (TeBeest et al. 2007) causing up to 30% of crop loss annually, thereby threatening global food security (Sakulkoo et al. 2018). Outbreak of this disease is a serious concern in many Asian and African countries, where the yield loss under epidemic conditions ranged from 60% to 100% (Kihoro et al. 2013). Further, it is estimated that the yield loss caused by this pathogen every year globally is equivalent to quantity required feed 60 million people (Barman and Chattoo 2005). Exploiting the host plant resistance is the viable and most preferred option to manage this disease as the diverse germplasm with wide variation for resistance is available. To date, over 100 blast resistance genes (Zhao et al. 2018) and 350 QTL were mapped on rice chromosomes (Singh et al. 2015; Ma et al. 2015) and 67 such *Pi* genes were designated (Xinga et al. 2015; Srivastava et al. 2017). Dominant R genes offer vertical resistance which is race-specific and prone to rapid breakdown in nature leading to “boom and bust” scenario. On the other hand, field resistance contributed by QTL offer horizontal or partial resistance, which is known for its durability in combating the disease out-breaks in a race non-specific manner (Fukuoka et al. 2015).

Wild species of *Oryza* and landraces are reservoirs and donors for genetic variability (Arbelaez et al. 2015), which needs to be explored for the discovery of novel gene(s) conferring tolerance to biotic stresses. As on date, only two blast resistance genes (*Pi9* and *Pi40*) were identified from wild species i.e. *O. minuta* and *O. australiencis*. However, a total of 350 QTL were identified for blast resistance, which includes QTL for field resistance (Ballini et al. 2008; Yan et al. 2017). The gene *Pi25(t)* derived from the cultivar Gumei-2 is reported to confer resistance to both leaf and neck blast (Zhuang et al. 2002). Another gene, *Pi-jnw1*, derived from a *japonica* landrace *Jiangnawqnwan* confers resistance to neck and leaf blast (Wang et al. 2016). Recently, QTL conferring field resistance to both the stages of blast was reported in an *indica* landrace Akhanaphou (Aglawe et al. 2017). Although a significant number of QTL were identified for blast resistance, only two of them i.e. *pi21* and *pi66* were cloned and characterized (Fukuoka and Okuno 2001; Wang and Pan 2016), which signifies a time consuming and technically demanding process involved in delimiting QTL to a single gene (Liu et al. 2013). However, the availability of genomic and phenomics resources enables the prediction of QTL more accurately, aiding in their characterization. Recent mapping studies have successfully illustrated the acceleration of breeding program through traditional mapping coupled with genomic approaches (He et al. 2014; Parida et al. 2017). Here, we report the identification of a large effect QTL conferring field resistance to leaf and neck blast; another QTL for leaf blast resistance using different mapping populations developed from a unique donor (which was registered as novel genetic resource for blast resistance; Rama Devi et al. 2015; Singh et al. 2016). Restriction-site associated DNA (RAD) sequencing was employed for the fine mapping of large effect QTL, *qBL3*. By combining mapping and microarray analysis, a putative candidate gene coding for malectin domain containing serine threonine kinase was identified in the mapped region. The effect of identified QTL, *qBL3* was validated by precisely transferring it into a susceptible variety through marker-assisted backcross breeding (MABB) and the best progeny lines exhibiting blast resistance was identified. Sequence analysis of alleles (promoter and its coding part) of candidate gene from resistant and susceptible lines revealed the presence of unique SNPs at coding as well as promoter region. The candidate gene linked markers identified along with improved lines possessing QTL for blast resistance developed through this study are novel genomic and genetic resources, which can be effectively used for imparting blast resistance in rice improvement programs.

## Methods

### Plant Material and Mapping Populations

An introgression line INGR15002, derived from the cross PR114/*O. glumaepatula* (IRGC 104387)//2*PR114, was identified and registered as a donor for field resistance to leaf and neck blast (Rama Devi et al. 2015; Singh et al. 2016). It was crossed to BPT5204 (Samba Mahsuri), a mega rice variety popular for its eating quality and CO39, a susceptible variety for leaf blast to develop different mapping populations viz., INGR15002/BPT5204 (F_2_ & F_3_), INGR15002/BPT5204/*BPT5204 (BC_1_F_2:3_), INGR15002/CO39 (F_2_) (Table 1).

**Table 1:** Summary of QTLs identified across different mapping populations with ICIM software

Mapping population	Population	Population size	Chrm	Marker intervals	LOD	R^2^	AE	DE
1. INGR15002 / BPT 5204	F_2_	188	3	RM14635(6.8) – RM14761(9.8)	11.7	34.7	−0.89	−0.08
			7	RM21052(3.7) – RM 1377 (12.0)	8.6	25.5	−0.83	0.01
	F_3_	188	3	RM14635(6.8) – RM251(10)	11.7	34.8	−0.89	−0.14
			7	RM21052(3.7) – RM 1377 (12.0)	8.5	25.4	−0.83	−0.02
2. INGR15002 / BPT 5204 (NE_Leaf)	F_2_	188	3	RM14635(6.8) – RM251(10)	10.9	28.8	−1.11	0.33
	F_3_	188	3	RM14635(6.8) – RM251(10)	10.8	28.7	−1.11	0.33
3. INGR15002 / BPT 5204 (NE_Neck)	F_2_	188	3	RM14635(6.8) – RM251(10)	9.1	32.7	−1.07	−0.16
	F_3_	188	3	RM14635(6.8) – RM251(10)	8.7	32.6	−1.02	−0.01
4. INGR15002 / CO-39	F_2_	188	3	RM3195(4.4) – RM251(10)	11.6	35.5	35.5	−1.1
			7	RM21052(3.7) - RM1377 (12.0)	5.9	14.9	14.9	−0.7
5. INGR15002 / BPT5204//BPT 5204	BC_1_F_2:3_	188	3	RM14635(6.8) – RM14761(9.8)	9.6	30.7	−1.08	0.19
			7	RM21052(3.7) – RM1377(12.0)	7.5	22.8	−0.98	−0.02

Numbers in parenthesis are the physical positions of the markers

*Chrm* Chromosome, *LOD* Logarithm of Odds, *AE* Additive Effect, *DE* Dominant Effect, *R*^*2*^ Phenotypic Variance

### Evaluation of Leaf Blast Resistance

Four mapping populations along with the parents and susceptible check HR-12 (used for leaf blast evaluation) were sown on uniform blast nursery beds at ICAR-Indian Institute of Rice Research (ICAR-IIRR), Hyderabad. After 15 days of germination, they were screened for leaf blast using a mixture of seven virulent isolates (to simulate the field resistance) collected from India as well as at field conditions at North East India (Aglawe et al. 2017; Madhav et al. 2013) (Table S1A). The data on leaf blast were recorded on 0–9 scale of Standard Evaluation System (SES) as described by International Rice Research Institute, Philippines (IRRI 2014). In the F_2_ population, blast score was recorded on individual plants, whereas mean blast scores of 20 plants in each F_3_ family were used for data analysis.

### Screening for Neck Blast Resistance

For neck blast, the F_2_ and F_3_ populations (INGR15002/BPT5204) were evaluated under natural conditions at Barapani (Manipur) in North Eastern region of India. Sowings were done in two sets - one set was used for leaf blast and another set for neck blast. To obtain a natural high neck blast incidence, three panicles for each F_2_ and BC_2_F_2_ plant were syringe inoculated with a mixture of four isolates collected from the hotspot region of NE India (Table S1B). In case of F_3_ and BC_2_F_3_ progeny, a set of three plants per family and three panicles per plant were syringe inoculated. To achieve a high and homogenous disease incidence, a susceptible check was planted for every five entries. Each plant under evaluation was scored three times in order to record for disease severity and the highest score was considered for analysis. The severity of neck blast was recorded as percentage of infection on the neck of rice panicle at physiological maturity as described by Madhav et al. (2013).

### Marker Analysis

Genomic DNA of all the individuals generated from different crosses was isolated using modified CTAB method (Saghai-Maroof et al. 1984). Polymorphism survey among the parents was carried out using a set of 926 SSR markers (McCouch et al. 2002) covering the 12 linkage groups of rice. PCR and electrophoresis protocols were performed as per Umakanth et al. (2017). For QTL mapping, a set of 150 polymorphic SSR markers distributed across 12 linkage groups were used initially.

### Linkage Analysis and QTL Mapping

A linkage map was constructed using the genotyping data of 150 polymorphic SSR markers. Phenotypic data collected from different crosses at two different locations (Table 1) and the genotypic data derived from F_2_ (*n* = 188; INGR15002/BPT5204; INGR15002/CO39) and BC_1_F_2_ (*n* = 188; INGR15002/BPT5204//BPT5204) population were used for QTL analysis. To detect the QTL, the software, inclusive composite interval mapping (ICIM) version 4.0 with additive and dominant QTL (ICIM-ADD) methods was chosen (http://www.isbreeding.net). Parameters like LOD score of > 5.0, window size of 1 cM, 1000 permutations and type I error set at 0.05 were applied for the QTL identification.

### Identification of SNP Markers and Fine Mapping

A modified version of RAD sequencing known as ddRAD was performed in two parents (INGR15002 and PR114) using Illumina TrueSeq chemistry on Illumina Hi Seq 2000 (Peterson et al. 2012). An additional set of F_2_ mapping population (*n* = 376; INGR15002/BPT5204) was used for the fine mapping studies. Also, additional polymorphic SSR and SNP markers (Table S2a) were used for the fine mapping of QTL. SNP genotyping was performed through allele specific primers.

### Microarray Analysis

The resistant (INGR15002) and susceptible (PR114) lines were grown in a growth chamber maintained at 26 °C with 70% relative humidity with 14 h day and 10 h night regime. The mock samples were inoculated with water while the test samples were spray inoculated with blast inoculums as described earlier. The leaf tissues were collected before and after infection at 10 dpi. Total RNA was isolated using the protocol described by Chomczynski (1993). Labeling, microarray hybridization and scanning were followed using the method described by Pallavi et al. (2016). The raw data obtained was analyzed using Agilent Gene Spring GX software (http://www.genomics.agilent.com/en/Microarray-Data-Analysis-Software) as described by Sinha et al. (2017). Differentially expressed genes were clustered using hierarchical clustering based on Pearson coefficient correlation algorithm to identify significant expression patterns. Genes were classified based on functional category and pathways using Genotypic Biointerpreter - Biological Analysis Software (http://genotypic.co.in/Products/4/Biointerpreter.aspx).

### qRT-PCR

*qRT-PCR* was performed among INGR15002; PR114 and BPT 5204 [was added as a susceptible check for comparisons and the tissue samples were collected as discussed earlier]. cDNA was synthesized using Thermo cDNA synthesis kit as per manufacturer’s instructions. Triplicate quantitative assays (three biological replications and for each biological replication three technical replications) were performed using 1 μl cDNA dilution samples with SYBER Green Master Mix (SYBR® Premix Ex Taq™ II, Tli RNase H Plus, RR820A) in Light Cycler® 96 Real-Time PCR. The primers for different genes were designed using Primer 3 (Table S2b). Actin was used as internal control to normalize data. To evaluate the quantitative variation between the samples, a relative quantification method was followed using Light Cycler® 96 SW 1.1 software. Microsoft excel was used to determine the correlation between the microarray data and the real-time gene expression data.

### Gene Cloning and In Silico Identification of TFBMs

The candidate gene (along with the promoter) in the fine mapped region was cloned from four parents (INGR15002; BPT5204; PR114 and CO39) using gene-specific primers. cDNA was cloned using another set of primers (Table S2c). PCR products were amplified using hi-fidelity *Taq* polymerase (MBI Fermentas) and cloned separately into pGEM-T-Easy vector of 3 kb using JM109 competent cells (Promega, USA). The positive transformants were selected and confirmed through PCR and restriction digestion with *Not I* enzyme. About 3–4 confirmed clones per amplicon were sequenced through Sanger sequencing (Eurofins, Bangalore). Based on the reference gene (Nipponbare) sequence and cDNA sequence information, promoter and genic regions were identified. For gene prediction, the online tools such as Genescan (http://genes.mit.edu/GENSCAN.html), Fgenesh (http://www.softberry.com) and Megante (https://megante.dna.affrc.go.jp/) were used. Conserved domains were identified through NCBI (https://www.ncbi.nlm.nih.gov/Structure/cdd/wrpsb.cgi). Nucleotide level polymorphisms among the alleles were identified by using Clustal Omega; MEGA 4.0 (http://www.megasoftware.net/) and DnaSP 5 (http://www.ub.edu/dnasp/) as reported earlier (Ramkumar et al. 2014). The transcription factor binding motifs (TFBMs) were identified using PlantPan 2.0 (http://plantpan2.itps.ncku.edu.tw/) and PLACE (http://www.dna.affrc.go.jp/PLACE/).

### Protein Localization and Modifications

Protein localization was predicted using the Protcomp software (http://www.softberry.com/berry.phtml?topic=proteinloc&prg=ProtCompA) with default parameters. Phosphorelation sites among the four sequences were analyzed using the online tool Musite (http://musite.net/).

### Introgression of qBL3 into Elite Rice Cultivar BPT5204

A BC_1_F_2_ line (IL-31–2-425) possessing the q*BL3* locus in homozygous condition selected based on the foreground selection using the linked markers was crossed with BPT5204 so as to develop BC_2_F_1_ and subsequently BC_2_F_2_. About 800 BC_2_F_2_ plants were used for phenotyping and subsequent development of BC_2_F_3_ homozygous progenies. Foreground selection was carried out with linked markers as shown in Table 1. The selected positive plants at each backcross stage were subjected to phenotypic selection using seven isolates at IIRR and four isolates at Barapani (Manipur) as per the method followed for the mapping of QTL. A set of 50 BC_2_F_3_ families that were resistant to leaf blast at IIRR and neck blast at NE were transferred to field and evaluated for agronomical traits. Background selection was performed using 125 SSR markers to determine the recovery of recurrent parent genome*.* Morphological features such as plant height, number of productive tillers, grain type, days to 50% flowering and average panicle length were recorded for the lines possessing the QTL in field condition for two seasons.

### Validation of QTL Linked Markers in Elite Rice Germplasm

The flanking markers of *qBL3* and *qBL7* were used to evaluate the polymorphism among the nine elite varieties, which are being used regularly in the rice breeding programs but susceptible to leaf / neck blast. The varieties tested are; Akshyadhan is a medium duration variety released for south India. Jaya (long bold) and Rasi (medium bold, drought tolerant, suitable for rain fed upland condition) are medium duration popular high yielding varieties released for North India. RP Bio-226 is a bacterial leaf blight resistant indica rice cultivar widely cultivated in South India and known for its high yield, premium grain quality, and excellent cooking qualities. Jarava has good yield traits and released for shallow lowland, coastal saline and irrigated ecosystem. MTU 1010 is one of the very popular mega-rice varieties of India, which is extensively cultivated across India. It has high yield, short duration and desirable long slender grain type. MTU 1081 and MTU 1153 are also short duration, non-lodging and high yielding varieties. Swarna is the popular rice variety mostly grown in the low land areas with high production potential.

## Results

Our earlier studies, have demonstrated that the introgression line (INGR15002) exhibited resistance to leaf and neck blast (Rama Devi et al. 2015; Singh et al. 2016). The present study focuses on understanding the genetics of resistance and identifying the gene(s) underlying the resistance mechanism.

### Mapping QTL for Field Resistance to Leaf Blast

**Fig. 1:**
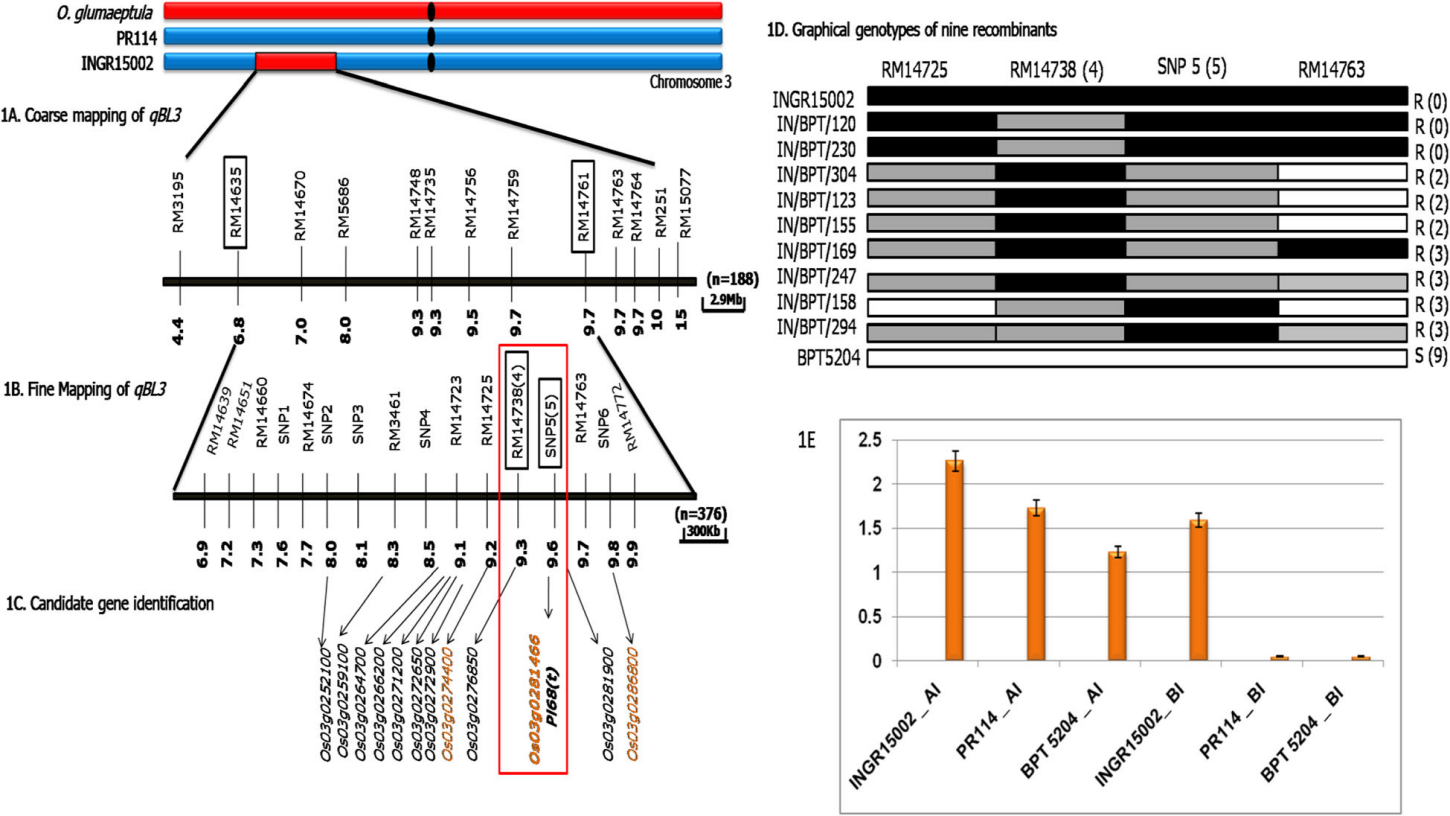
**a** The schematic representation of coarse mapped *qBL3* region. The markers were arranged based on their physical position in comparison with Nipponbare genome (The physical positions of the markers were retrieved the Gramene – Supplementary table 18 of class I SSRs (https://archive.gramene.org/markers/microsat/). **b** The schematic representation of fine mapped *qBL3* region. The numbers in parenthesis indicates the number of recombinants. The markers in encircled are the linked markers. **c** The gene *Os03g0281466* within the fine mapped region which is up-regulated in microarray based transcriptome studies and validated through *q*PCR. Of the 12 up regulated genes in microarray, only three genes are consistent with *q*PCR (indicated by Red color). The gene *Os03g0281466* is the only gene which is up-regulated in both microarray and *q*PCR and is falling with in the fine mapped region of *qBL3*. **d** Graphical genotypes of recombinants for the markers RM14738 and SNP5. R- resistant; S - Susceptible. The number in parenthesis is the phenotype score in SES (0–9) scale. The number on the left are the plant entry number. **e** Bar graph demonstrating the fold change difference in *Os03g0281466* which is induced in INGR15002 upon pathogen infection in *q*PCR

Segregation ratios of F_2_ population (developed from INGR15002 and BPT5204) for blast resistance showed continuous distribution, indicating the resistance in INGR15002 is quantitative in nature (Figure S1A). The phenotypic results of F_2_ were consistent with the F_3_ progeny test. Survey of polymorphism among the parents using 926 SSR markers led to the identification of 308 polymorphic markers (33.2%). Out of these, 150 polymorphic markers distributed equidistantly (~ 1 marker per 5 Mb) across the genome were selected for single plant analysis. Genotyping of 188 F_2_ individuals with 150 polymorphic markers resulted in a linkage map with a total length of 1364.99 cM and an average genetic distance of 9.09 cM between two markers (Figure S2a; S2b & S2c). Subsequent QTL analysis resulted in the identification of two QTLs, *qBL3* within the marker interval RM14635 - RM14761 on chromosome 3 (Fig. 1a); and another QTL, *qBL7* within the marker interval RM21052 - RM1377 on chromosome 7. The QTL identified were significantly associated with leaf blast resistance accounting for 34.7% and 25.5% of phenotypic variance, respectively with logarithmic of odds (LOD) score > 5.0 (Figure S3). The mapping results were consistent with F_3_ progeny data.

To check the consistency of the identified QTL, an alternate F_2_ population (INGR15002 crossed with CO39) was used (Figure S1B) and QTL analysis was done with another set of 150 polymorphic SSR markers. This led to the identification of the QTL, *qBL3* within the marker interval RM3195 – RM251 on chromosome 3, explaining 35.5% of the phenotypic variance to the leaf blast resistance. In addition, another QTL, *qBL7* was detected within the maker interval RM21052 – RM1377 on chromosome 7, contributing 14.9% of the phenotypic variance. The QTL results were also verified using a backcross population (*n* = 188; developed by crossing INGR15002 and BPT5204) and consistent results were observed (Table 1).

### Mapping QTL for Field Resistance to Neck and Leaf Blast at Second Location

North Eastern (NE) region of India, which is a hotspot for neck and leaf blast, was chosen as the second location for the determination of QTL. Phenotypic data of leaf and neck blast resistance was generated from Barapani (NE India) with two sets of same F_2_ individuals (Set 1 with plant numbers 1 to 188; Set 2 with plant numbers 189 to 377; *n* = 188) derived from a cross between INGR15002 and BPT5204 (Figure S1-C & S1-D). Genotyping was done using 150 polymorphic SSR markers. QTL analysis using the genotypic and phenotypic data resulted in the identification a QTL, *qBL3* on chromosome 3, within the marker interval RM14635 - RM251 explaining 28.8% and 32.7% of the phenotypic variance for leaf and neck blast resistance, respectively. But, the QTL, *qBL7* was not identified in this location. The mapping results were consistent with F_3_ progeny data (Table 1).

### Fine Mapping of the Major QTL

Linked markers of the QTL, *qBL3* were BLAST-searched against the reference genome (Nipponbare) resulting in the location of the linked marker RM14635 on 6.8 Mb position and another linked marker RM14761 on 9.7 Mb position on chromosome 3, thus corresponds to 2.9 Mb genomic region of Nipponbare. Forty SSR markers from the 2.9 Mb region were used for polymorphism assay between the parents and found ten markers were polymorphic. Hence, ddRAD sequencing was employed for the identification of SNPs. A total of 6649 SNPs were identified between the parents (INGR15002 and PR114) (Table S3). Among the 51 SNP’s present at *qBL3* region, 15 high quality SNPs were (present in read depth of 10 sequences) selected for genotyping which resulted in to the identification of polymorphism of six SNPs between INGR15002 and BPT5204. These polymorphic SNPs and SSRs together genotyped in the large population (INGR15002/BPT5204 F_2_; *n* = 376) (Figure S1E), which resulted in the fine mapping of *qBL3* within the marker interval RM14738 - SNP5 having phenotypic variance of 52%. (Figure S4 and Table S4). Thus the physical distance of the QTL was narrow down to 300 Kb [RM14738 (9.3 Mb) and SNP5 (9.6 Mb)] from 2.9 Mb (Fig. 1b & d).

### Identification of Candidate Gene

To identify the differentially expressed genes between two parents (INGR15002 and PR114) before and after infection, a microarray analysis was carried out with various combinations. A total of 722 genes were up-regulated in INGR15002 after infection as compared to PR114 (Figure S5 & Table S5). From the fine mapped *qBL3* region of 300Kb, only one gene i.e. *Os03g0281466* was present which is specifically induced in resistant parent after infection (Fig. 1c). The common and unique genes among the parents that were up and down-regulated in various datasets within the coarse mapped *qBL3* region were represented as Venn diagram (Figure S6 and S7 and Tables S6 and S7). In addition to *Os03g0281466*, up-regulation of many transcripts related to transport, ATP binding, transcription, stress, cell wall, signaling and secondary metabolisms were found in the resistant parent INGR15002 (Figure S8). Similarly, DEGs in *qBL7* and down-regulated genes were also found and tabulated in Table S8 and S9.

### Validation of Expression

Top three up-regulated genes from the coarse mapped region of *qBL3* and top six up-regulated genes from *qBL7* mapped regions, were selected for *qRT*-PCR analysis. Among those genes, up- regulation of *Os03g0281466* related to resistance was clearly observed in INGR15002 after infection in microarray and *qRT*-PCR (Fig. 1e). The expression patterns of selected genes in *q*PCR were consistent with the microarray exhibiting a significant correlation (*R*^*2*^ = 0.77) (Table S10).

### Cloning of cDNA, Gene and Promoter of *Pi68(t)*

By integrating fine mapping, transcriptome and *q*PCR data, *Os03g0281466* has been identified as a promising candidate gene in imparting leaf and neck blast resistance in INGR15002 and the gene is designated as *Pi68(t).* Sequence of the candidate gene (*Os03g0281466*) from the resistant parent (INGR15002) was used for the prediction of gene structure through various software’s as discussed in Methods section. The consistent results obtained have revealed that the gene has a single ORF of 2519 bp encoding 839 amino acids. The sequence of the cloned cDNA of the resistant line showed 100% identity to the predicted ORF of the candidate gene. The deduced protein has two major domains - malectin domain (from 35 to 396 amino acids) and catalytic domain of serine threonine kinase (STKC-IRAK) (from 515 to 776 amino acids). The catalytic domain consists of overlapping ATP binding site (from 515 to 649 amino acids), activation loop and polypeptide binding site (650–676 amino acids). The protein *Pi68(t)* is a plasma membrane localized protein with trans-membrane domain as predicted by the Protcomp software.

### Allelic Variations of *Pi68(t)*

**Fig. 2:**
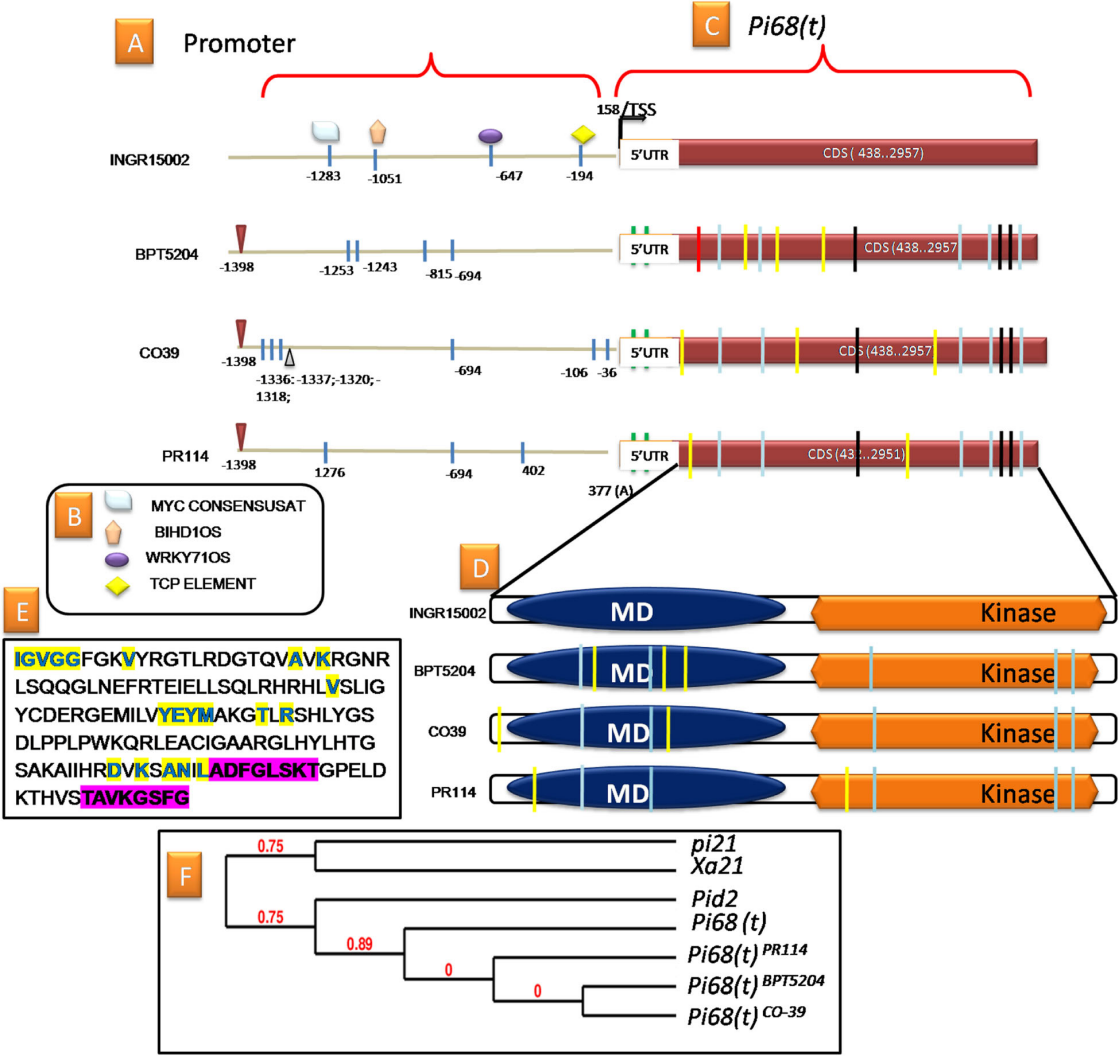
Promoter and gene characterization of *Pi68(t)* alleles in resistant and susceptible lines. **a** The number written in the promoter region indicates the position of SNPs identified in each sequence. The SNP leading to the presence/absence of TFBM was shown using different shapes. The triangle (Black) in CO39 promoter indicates the single base pair deletion and the triangle (red) indicate single base pair insertion. **b** The list of TFBMs identified in the promoter region and function of each motif is given in Table S12. **c** The TSS and coding sequence of *Pi68.* The two SNPs exist in the 5’UTR region were indicated in black bars. The blue bars in the coding sequence region indicate the common SNPs in the susceptible alleles leading to non synonymous changes. The black bars in the coding sequence region represent the common SNPs in the susceptible alleles leading to synonymous changes. The yellow bars in the coding sequence region are the allele specific SNPs leading to non synonymous changes. The red bars in the coding sequence region are the allele specific leading to synonymous changes. The position of the SNP and its Synonymous and Non Synonymous substitutions of amino acid are tabulated in Table S11. **d** The functional domains of the predicted protein. **e** The signature sequences of the active and ATP binding sites were highlighted in yellow and the signature sequences of Activation Loop and polypeptide binding site were highlighted in pink. **f** Cladogram representing the relationship of the *Pi68* with earlier reported kinases of rice blast (*pi21; Pid2*) and bacterial blight (*Xa21*)

All the susceptible alleles have five common SNPs (two were in the malectin domain and three were in the kinase domain) leading to a non-synonymous amino acid (AA) change in the protein. These amino acid change in malectin and kinase domain in the susceptible protein (at 165 T/A, 303 D/N; 686 I/M, 715 S/P, 761 Y/C) might play a role in conformational changes, which might be responsible for lack of resistance. For instance, the presence of Threonine (T) at position 165 in malectin domain is involved in formation of the kinase-specific phosphor-serine-threonine (Protein kinase B), which was replaced by Alanine (A) in the susceptible protein. In addition to the common non-synonymous amino acid changes, there were sequence-specific non-synonymous amino acid changes present in BPT5204 (3), PR114 (2) and CO39 (2) proteins (numbers in parenthesis indicate the number of non-synonymous amino acid changes in each sequence). Furthermore, two specific variations at 5′ UTR [162 (C/T) and 402 (G/A)] were also identified among the susceptible alleles. The complete details of the SNPs and the corresponding synonymous and non-synonymous changes are tabulated in Table S11 and Fig. 2.

Key variations (five SNPs) were found at the promoter regions of all susceptible alleles, of which, four SNPs led to the absence of four important TFBMs i.e. W-Box element, MYC element; BIHD1OS (benzothiadiazole-induced homeo domain protein); and TCP element. The resistant allele has all the four motifs which might play an important role in the resistance mechanism (Table S12). Among the analyzed alleles, Ka/Ks ratio was least for *Pi68(t)*^*BPT5204*^ indicating that this allele has least evolutionary deviation compared to the other two susceptible alleles. The complete sequence of the gene along with promoter of all the alleles studied were submitted to NCBI with accession numbers MG742320; MG742321; MG742322; MG742323.

### Validation of qBL3 through Marker-Assisted Backcross Breeding

**Fig. 3:**
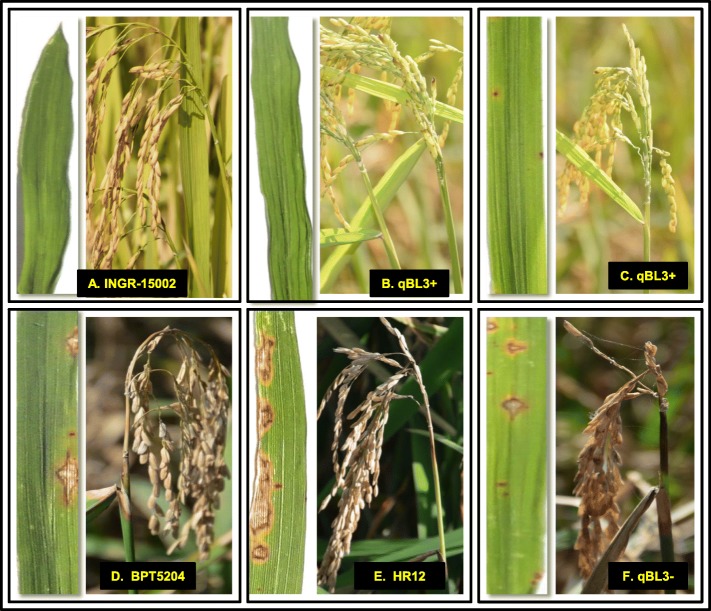
Phenotypic evaluation of parent (**a** INGR15002); BC2F3 promising entries (**b** & **c** having *qBL3;* plant numbers, 31 R & 78 R); along with susceptible checks (**d** BPT5204; **e** HR12); **f** is the BC2F3 line that does not have *qBL3*; the plants containing *qBL3* showed better response than the susceptible checks BPT5204 & HR12. The right panel of each picture corresponds to leaf blast and left panel to neck blast

To know the effect of major QTL in imparting resistance, *qBL3* was introgressed into Samba Mahsuri (BPT5204; highly blast susceptible variety yet popular for its excellent grain and cooking quality) through marker-assisted backcross breeding (MABB) coupled with phenotyping. The complete details of the number of F_1_, BC_1_F_1_, BC_2_F_1_ plants raised and the corresponding positive plants identified at each stage were tabulated in Table S13 along with the number of markers tested. Among the 800 BC_2_F_2_ plants, about 400 were screened at IIRR for leaf blast, of which, 100 plants were homozygous positives. Among the other 400 BC_2_F_2_ plants screened at NE region, about 100 plants were homozygous positive for both leaf and neck blast resistance. Thus, a set of 200 homozygous BC_2_F_2_ plants were identified through foreground selection using markers linked to *qBL3* as well as through phenotyping. Initially, foreground selection was performed using linked markers (Table 1). However, after fine mapping, BC_2_F_3_ homozygous resistant plants were confirmed with the closely linked markers, SNP5 and RM14738, whose results were consistent. Fifteen homozygous BC_2_F_2_ plants (having *qBL3*) were selected for background selection analysis and the recovery of recurrent parent genome ranged from 93% to 97% (Table 2), which were advanced to BC_2_F_3._ The BC_2_F_3_ lines possessing *qBL3* have shown the leaf blast score of 1.1 to 2.1 at IIRR whereas the same lines have shown the leaf and neck blast scores ranging from 1.0 to 1.9; 1.2 to 1.8, respectively at NE region (Fig. 3 and Figure S9). Based on morphological traits and phenotypic scores, the best 15 lines having *qBL3* were identified (Figure S10 & Table S14). The usefulness of markers linked to the identified QTL was tested in a set nine elite lines, which are very popular for their yield, grain and cooking qualities but susceptible to blast (as discussed in material and methods). It was observed that, *qBL3* and *qBL7* linked markers were polymorphic among the tested entries and these markers could be efficiently used in blast resistance breeding programs (Table S15).

**Table 2:** Phenotyping and genotyping of *qBL3* BC_2_F_3_ lines

	# BC_2_F_3_ plant entry no.	Genotype with *qBL3*	Phenotype	^a^Leaf blast score (BC_2_F_3_ families)	^b^Leaf blast score (BC_2_F_3_families)	^b^Neck blast score	
						(BC_2_F_3_ families)	Percentage of background recovery at BC_2_F_2_
1	31_R	RR	R	1.1	1.0	1.2	97%
2	78_R	RR	R	1.1	1.7	1.0	97%
3	85_R	RR	R	1.1	1.6	1.2	97%
4	112_R	RR	R	1.4	1.0	1.3	97%
5	165_R	RR	R	1.1	1.1	1.2	97%
6	174_R	RR	R	1.4	1.5	1.3	95%
7	176_R	RR	R	2.1	1.2	1.5	95%
8	189_R	RR	R	1.2	1.0	1.8	95%
9	109_R	RR	R	1.1	1.1	1.6	95%
10	211_R	RR	R	1.3	1.9	1.5	95%
11	275_R	RR	R	1.1	1.5	1.8	93%
12	293_R	RR	R	1.2	1.9	1.6	93%
13	94_R	RR	R	1.3	1.5	1.6	93%
14	269_R	RR	R	1.4	1.8	1.8	93%
15	325_R	RR	R	1.2	1.8	1.8	93%
	INGR15002	RR	R	1.1	1.2	1.0	–
	BPT 5204	SS	S	9.0	9.0	9.0	–

Mean blast scores obtained from 10 plants of each BC_2_F_3_ families

^a^ at IIRR

^b^ at NE

## Discussion

Many studies in the past have proven the potential of wild species (*O. minuta and O. australiencis*) and landraces (Jiangnanwan; Akhanaphou) as donors for leaf and neck blast resistance (Amante-Bordeos et al. 1992; Jeung et al. 2007; Wang et al. 2016; Aglawe et al. 2017; Cho et al. 2008). The present study was designed to map the chromosomal regions associated with leaf and neck blast resistance from INGR15002 which has introgression region from *O. glumaepatula* on chromosomes 3 and 7 and does not contain the alleles of major blast resistant genes of India (Rama Devi et al. 2015). Evaluation for leaf and neck blast in the present study was carried out using a mixture of isolates under natural and artificial conditions at two different locations. Similar kind of evaluations were reported by Fang et al. (2019) using a single blast pathogen; Xiao et al. (2017) using mixture of isolates The resistance of neck blast in INGR15002 was verified by Singh et al. (2018), by screening with most virulent isolate (NB-7) at both natural as well as artificial conditions and recommended the line for blast breeding programs of India. The studies on pathogenicity of blast isolates also revealed variability in aggressiveness, suggesting a potential existence of different races at varied environments (Xue et al. 2012; Onaga et al. 2015). Till date, genes like *pi25(t), pb1* and *Pi64* (Zhuang et al. 2002; Hayashi et al. 2010; Ma et al. 2015) and QTL, *qPbm11* (Ishihara et al. 2014); *qNB1–1, qNB11–1, qNB1–2, qNB12–2, qNB1–3, qNB11–3, qNB12–3* (Noenplab et al. 2006) were identified for neck blast resistance. Of them, *Pi64* is the only dominant gene reported to be involved in leaf as well as neck blast resistance.

QTL analysis in INGR15002 resulted in the identification of two major QTL, *qBL3* and *qBL7* on chromosomes 3 and 7. Based on till date reports, chromosome 3 has a recessive gene *pi66* (from *aus* type cultivar AS20–1) near telomere (Wang and Pan 2016). Genome-wide association study (GWAS) using 366 diverse *indica* accessions reported 20 candidate genes on chromosome 3 (Chr03_1170958 locus) for rice blast (Wang et al. 2014). However, both loci were far away from *qBL3* of the current study. Furthermore, Wang et al. (2015) also reported 21 SSR markers associated with rice blast resistance using GWAS analysis. Of them, RM232 located at 9.75 Mb (is near but not within the fine mapped region of *qBL3)* is identified to be associated with blast resistance. Interestingly, *qBL3* is nearer to one of the loci associated with field blast resistance (LAFBRs) identified by Zhu et al. 2016. Hence *qBL3* is a novel QTL associated for leaf and neck blast resistance. In addition, Wang et al. (2015) reported RM125 (located at 5.48 Mb) associated with rice blast resistance using GWAS. This marker is present within the QTL region of *qBL7*. However, *qBL7* identified in the present study was mapped away from the *qPbh-7-1*, a QTL reported for panicle blast by Fang et al. (2016) as well as the QTL identified for leaf blast resistance by Sirithunya et al. (2002). Many reports demonstrated a cluster of blast R genes on chromosomes 6, 11 and 12 (Singh et al. 2015) indicating the importance of these chromosomes for conferring blast resistance. But, there is a need to explore the novel loci to address the everlasting arms race of host pathogen evolution.

The consistency and stability of any identified QTL was determined by use of alternate mapping populations and evaluating them at diverse environments as reported by Mishra et al. 2013. Advanced backcross mapping population (BC_2_F_2:3_) was used for identification of QTL and simultaneous introgression in to elite variety, similar strategy was reported Tanksley and Nelson 1996 and Robin et al. 2003. The inconsistent map locations between the leaf and neck blast resistance indicated the complexity of fixing both leaf and neck blast resistance. This is the first report on the identification of a major QTL, *qBL3* derived from the wild species *O. glumaepatula* for leaf and neck blast resistance.

The *qBL3* region was primarily targeted for fine mapping, since it was contributing resistance for leaf as well as neck blast. This facilitated in the rapid identification of putative candidate gene(s) associated with blast resistance. *qBL7* locus is quite larger, its contribution to leaf blast resistance is less compared to *qBL3* and it was not identified in the second environment, hence not chosen for further studies. To accelerate the fine mapping, ddRAD sequencing was employed between INGR15002 and PR114 since both are isogenic lines. Interestingly, a greater number of SNPs were observed on chromosomes 3 and 7 as compared to all other chromosomes, enabling fine mapping of the *qBL3* region from 2.9 Mb to 300 kb and identified SNP5 as tightly linked marker for *qBL3*. Our previous study (Rama Devi et al. 2015) also identified wild genome introgression at this locus, which is in support of our current results.

To identify putative candidate genes for *qBL3* and *qBL7,* integration of QTL mapping with microarray analysis was done. The parents (INGR15002 and PR114) are expected to have less variation since they are isogenic, except at few loci, hence the variation observed at transcript level would be primarily due to the wild genome introgression in INGR15002. Although BPT5204 was used as a susceptible parent in developing mapping populations, this parent was not included in the transcriptome analysis to confiscate obvious variations between INGR15002 and BPT5204. However, while performing the *q*PCR, BPT5204 was chosen as an additional susceptible check. Though many genes were up-regulated in INGR15002 after infection, only one gene (*Os03g0281466)* encoding malectin serine threonine kinase was up-regulated in the *qBL3* fine mapped region. In addition, the peak marker SNP5 also lies very near (50 kb) to the gene *Os03g0281466,* hence it was identified as the putative candidate gene. Up-regulation of transcript derived from this gene was also reported in Nipponbare leaf infection with blast fungus [GSE18361] (Marcel et al. 2010). Thus, microarray-assisted mapping was successful in identifying the putative candidate gene of the large effect QTL, *qBL3*. Although few transcripts were up-regulated in *qBL7* loci, it is difficult with the current information to delineate the putative candidate gene(s) for *qBL7*. However, the results obtained would certainly help in the fine mapping of *qBL7* locus in future.

The gene *Os03g0281466* was annotated as a hypothetical gene in the RAPDB database (coding for 213 amino acids). Cloning cDNA as well as predicting the ORF with three different algorithms confirmed the gene has single ORF and deducing the protein containing 839 amino acids with two domains viz, malectin and kinase domain. Since the origin of this gene is from wild species (*O. glumaepatula*), the change in the gene structure as well as protein is obvious. Serine threonine kinases were known to be involved in blast resistance mechanisms (Chen et al. 2006; Hurni et al. 2015). Till date, two kinase encoding genes *pi21* and *Pid2* were reported to be associated with rice blast resistance (Fukuoka and Okuno 2001; Chen et al. 2006). Malectin is a membrane-anchored protein of the endoplasmic reticulum that recognizes and binds Glc2-N-glycan. The domain is found in number of plant receptor kinases and known to play a role in defense mechanisms (Bellande et al. 2017; Rajaraman et al. 2016). We hypothesize that malectin transmembrane domain may receive the external cues in response to blast infection and trigger the signal transduction using the kinase domain. The up-regulation of transport, ATP binding, transcription, cell wall and stress-related genes in the resistant parent after infection strengthen our hypothesis that induction of *Pi68(t)* may trigger the downstream signal transduction pathways leading to resistance. However, this hypothesis needs further experimentation. The phylogenetic analysis of protein sequences of *Pi68(t)* clustered with the Pid2 as a separate clade, which signifies that they were grouped together as both genes encode for lectin (carbohydrate binding proteins). Nevertheless, *Pi68(t)* encode for malectin with kinase domain whereas *Pid2* encode for AB-lectin receptor kinase.

The nucleotide polymorphisms present in coding part of the gene has clearly differentiated the susceptible and resistant alleles. These changes might have led to protein conformational changes leading to susceptibility. The variations (SNPs) in 5’UTR as well as promoter region of all the susceptible alleles of *Pi68(t)* led to the absence of key TFBMs like W-box element, MYC element, TCP element and BIHD1OS, which may play a vital role in altering the gene expression. It is evident from previous studies that these elements are known to involve in defense mechanisms (Inoue et al. 2013; Deb and Kundu 2015; Thakur et al. 2015; Li 2015). Thus absence of these TFBMs in the promoter region might have led to susceptibility. The importance of promoter and its polymorphisms in resistance mechanisms were well reported (Ramkumar et al. 2014; Yuan et al. 2011).

We demonstrated the functional validation by precisely introgressing the QTL into the susceptible background of Samba Mahsuri (BPT5204) through MABB approach. Through evaluation of large (800 BC_2_F_2_ plants), best five lines with high recurrent genome recovery (97%) were identified and these lines showed resistance to leaf and neck blast at two hotspot locations. Further, these lines were nominated to All India Coordinated Research Project (AICRP) for studying their reaction towards field resistance at various locations across India. The simultaneous introgression and identification of superior lines with improved resistance would accelerate in releasing the variety, and reaching the farmers in a short time. The usefulness of identified linked markers in the present study were also assessed in the popular varieties (became susceptible to blast) so as to use them effectively in marker-assisted breeding programs (Kumar et al. 2011).

## Conclusions

The major outcomes of this investigation include the identification and high-resolution mapping of a major effect field resistance QTL from the wild species *O. glumaepatula*. Fine mapping coupled with expression profiling led to the identification of an inducible gene *Os03g0281466* (malectin serine threonine kinase) designated as *Pi68(t)* conferring resistance to leaf as well as neck blast. The SNPs located in the promoter, 5′ UTR and coding region might play a key role in the resistance mechanisms. QTL-NILs derived from Samba Mahsuri possessing the QTL, *qBL3* were identified, which exhibited high level of resistance to leaf and neck blast. The QTL linked functional markers and the MAS derived lines identified in this study will eventually aid in the building the durable broad-spectrum field resistance in race non-specific manner.

## Supplementary information

**Additional file 1.**

**Additional file 2.**

## Data Availability

Not Applicable

## Abbreviations

AA: Amino acid
DEGs: Differentially expressed genes
dpi: Days post inoculation
MABB: Marker-assisted backcross breeding
NILs: Near isogenic lines
QTL: Quantitative trait locus
R: Resistance
SNP: Single nucleotide polymorphism
SSR: Simple sequence repeats
TFBM: Transcription factor binding motif
