# Supplementary material for: Identification and Characterization of a Large Effect QTL from Oryza glumaepatula Revealed Pi68(t) as Putative Candidate Gene for Rice Blast Resistance
Source: Rice (N Y). 2020 Mar 12;13:17. doi: 10.1186/s12284-020-00378-4 (PMC7067966; doi:10.1186/s12284-020-00378-4)
Supplement: Supplementary file 1 — Additional file 1. [file 12284_2020_378_MOESM1_ESM.pptx]

## Slide 1
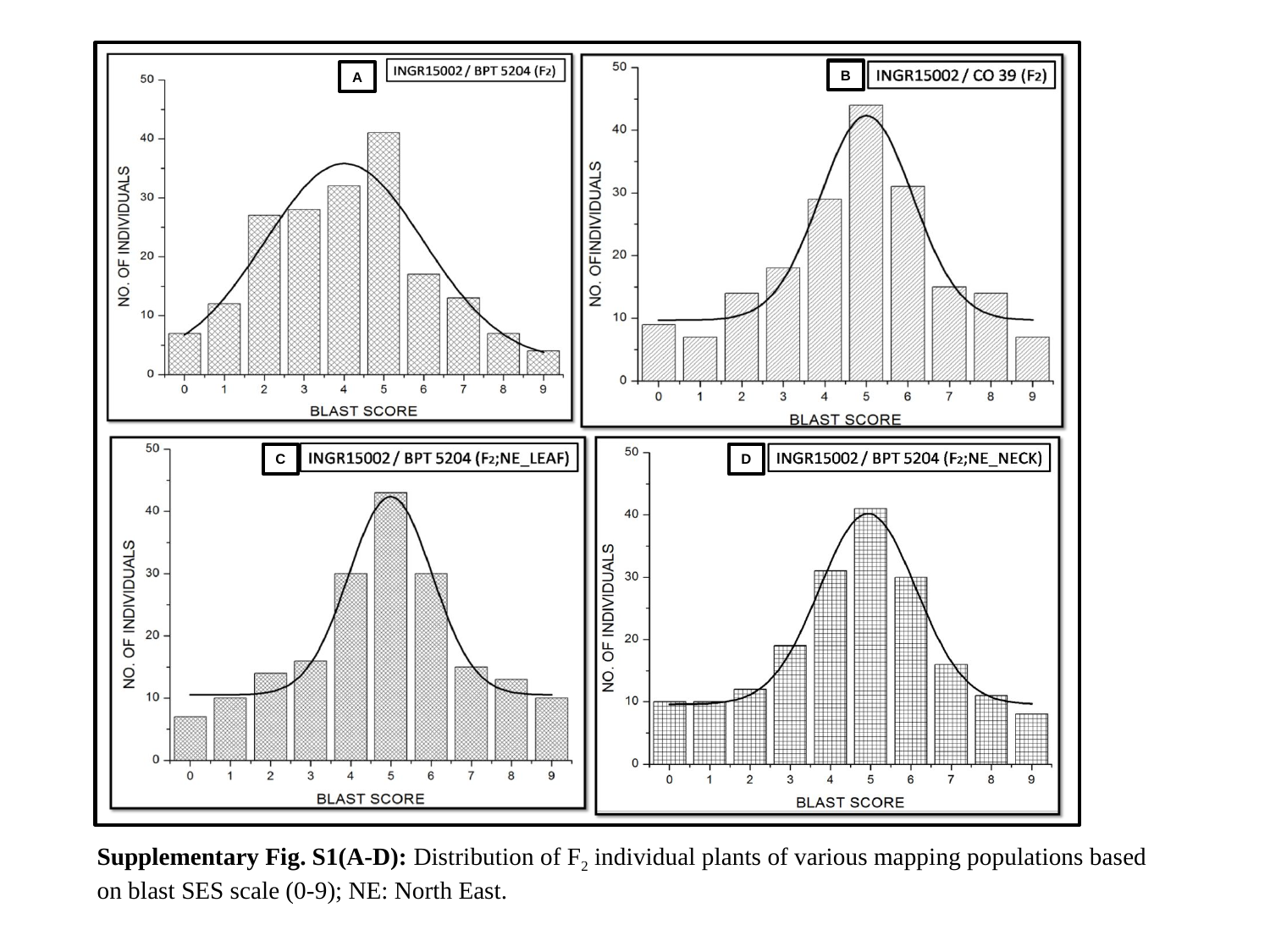

A
C
D
B
Supplementary Fig. S1(A-D): Distribution of F2 individual plants of various mapping populations based on blast SES scale (0-9); NE: North East.

## Slide 2
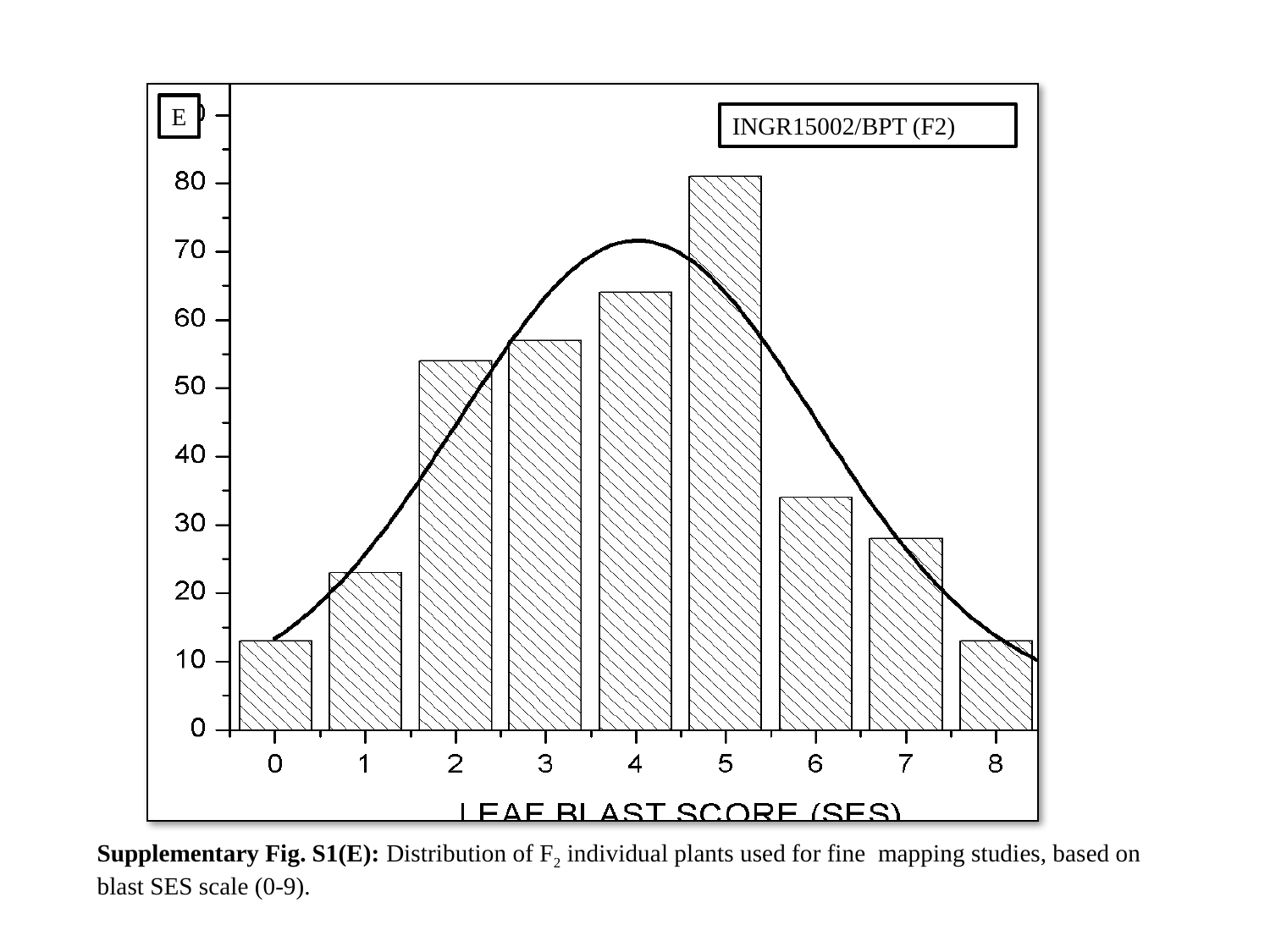

E
INGR15002/BPT (F2)
Supplementary Fig. S1(E): Distribution of F2 individual plants used for fine mapping studies, based on blast SES scale (0-9).

## Slide 3
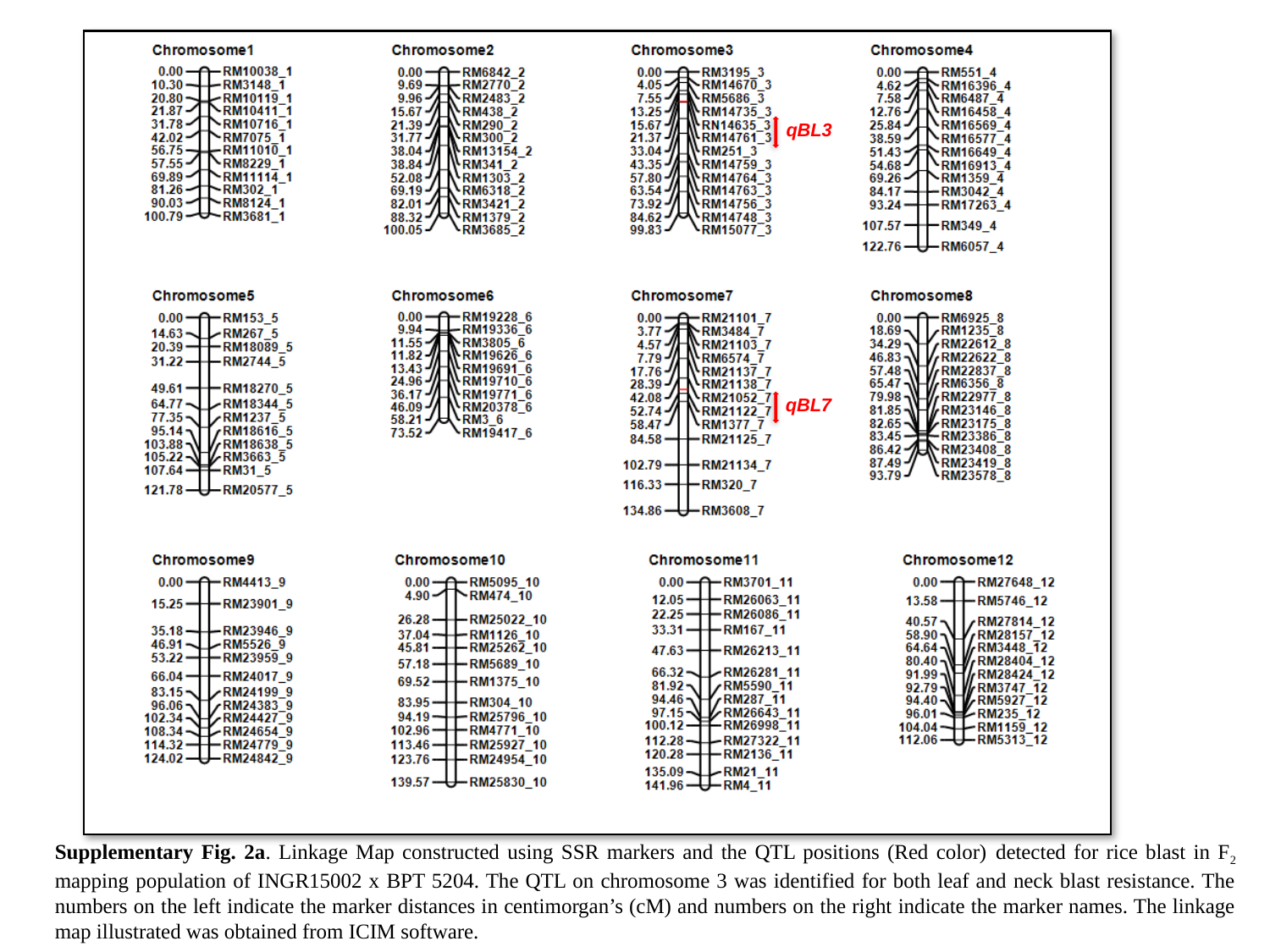

qBL3
qBL7
Supplementary Fig. 2a. Linkage Map constructed using SSR markers and the QTL positions (Red color) detected for rice blast in F2 mapping population of INGR15002 x BPT 5204. The QTL on chromosome 3 was identified for both leaf and neck blast resistance. The numbers on the left indicate the marker distances in centimorgan’s (cM) and numbers on the right indicate the marker names. The linkage map illustrated was obtained from ICIM software.

## Slide 4
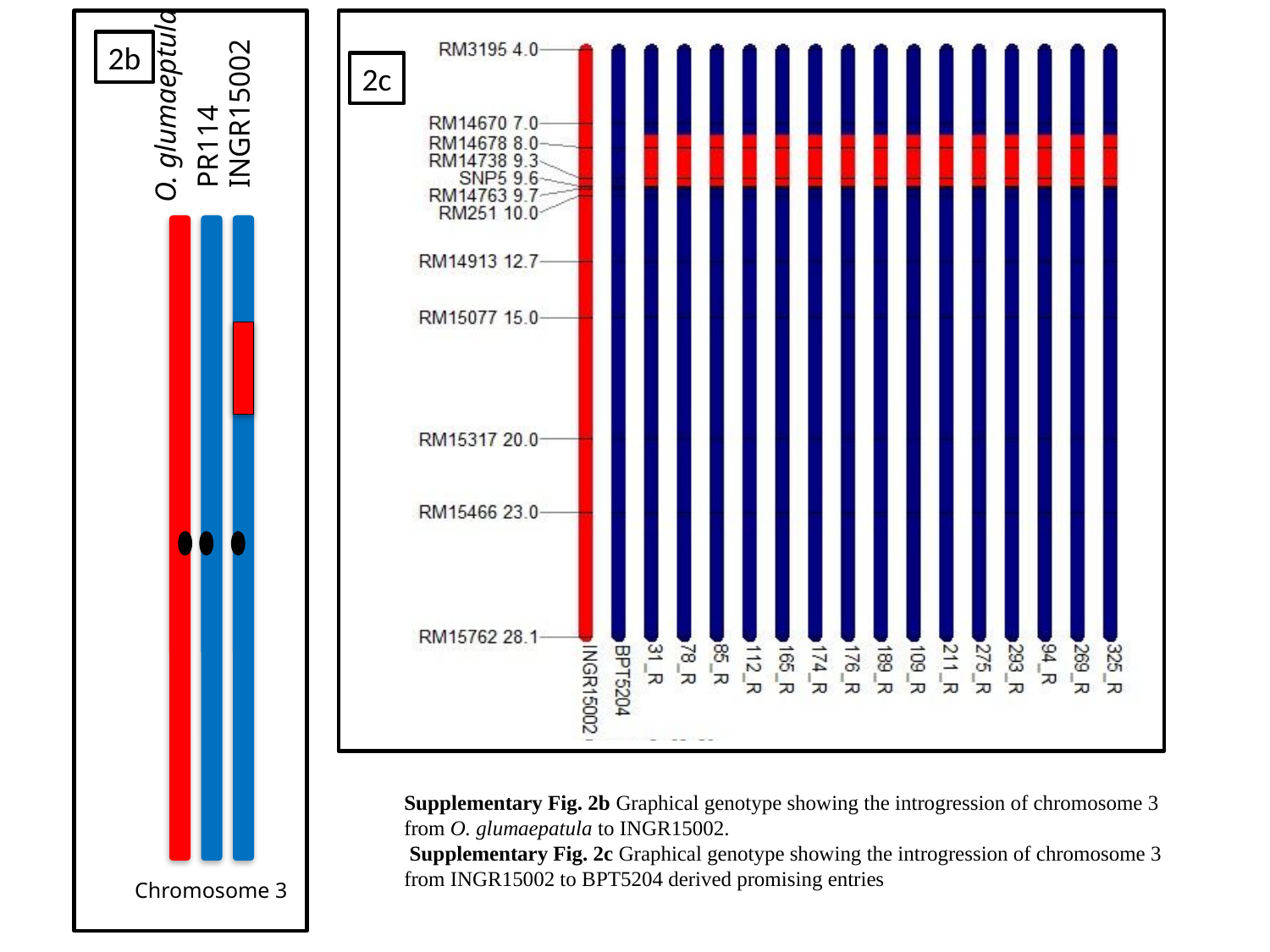

O. glumaeptula
INGR15002
PR114
Chromosome 3
2b
2c
Supplementary Fig. 2b Graphical genotype showing the introgression of chromosome 3 from O. glumaepatula to INGR15002.
 Supplementary Fig. 2c Graphical genotype showing the introgression of chromosome 3 from INGR15002 to BPT5204 derived promising entries

## Slide 5
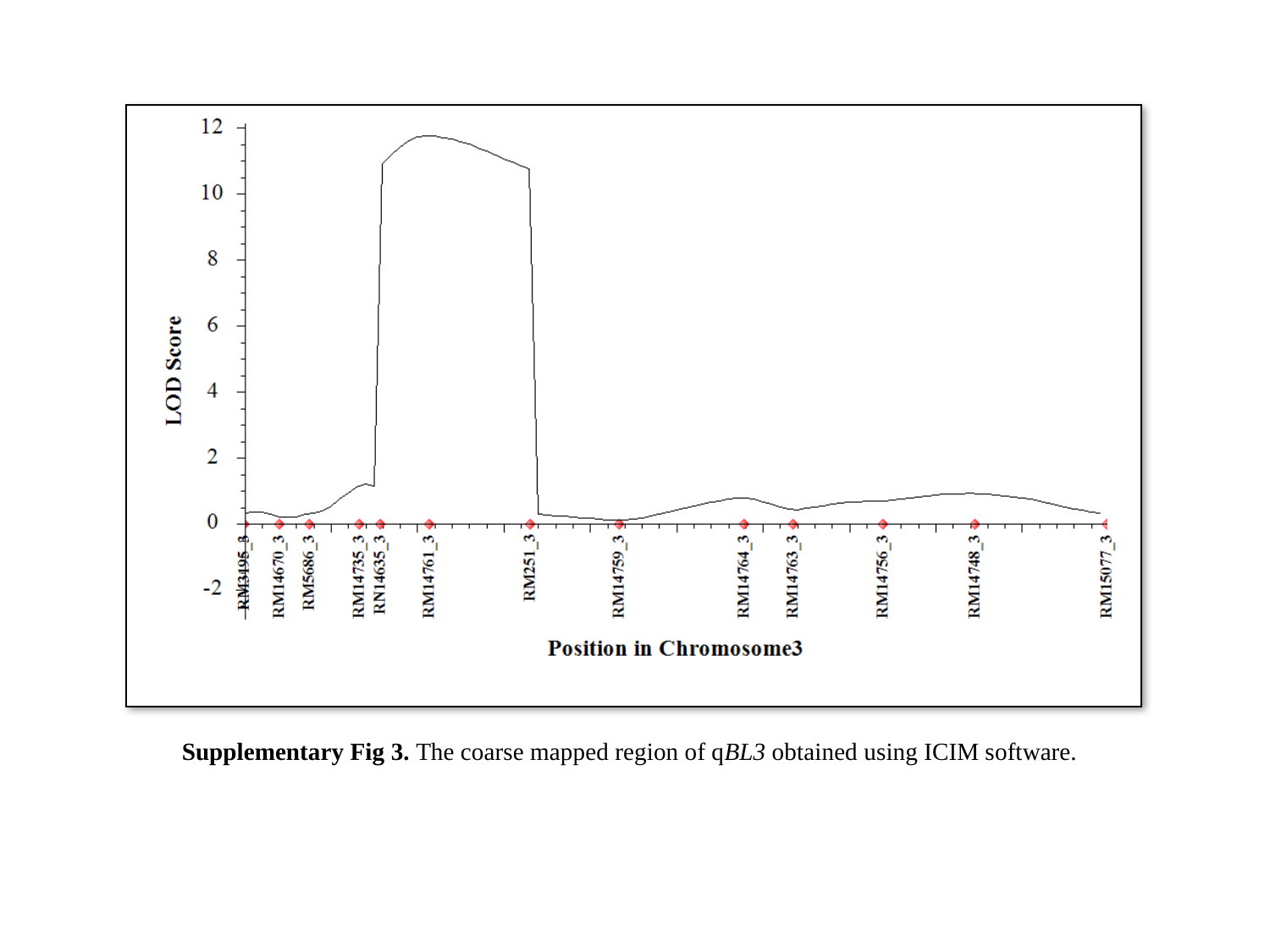

Supplementary Fig 3. The coarse mapped region of qBL3 obtained using ICIM software.

## Slide 6
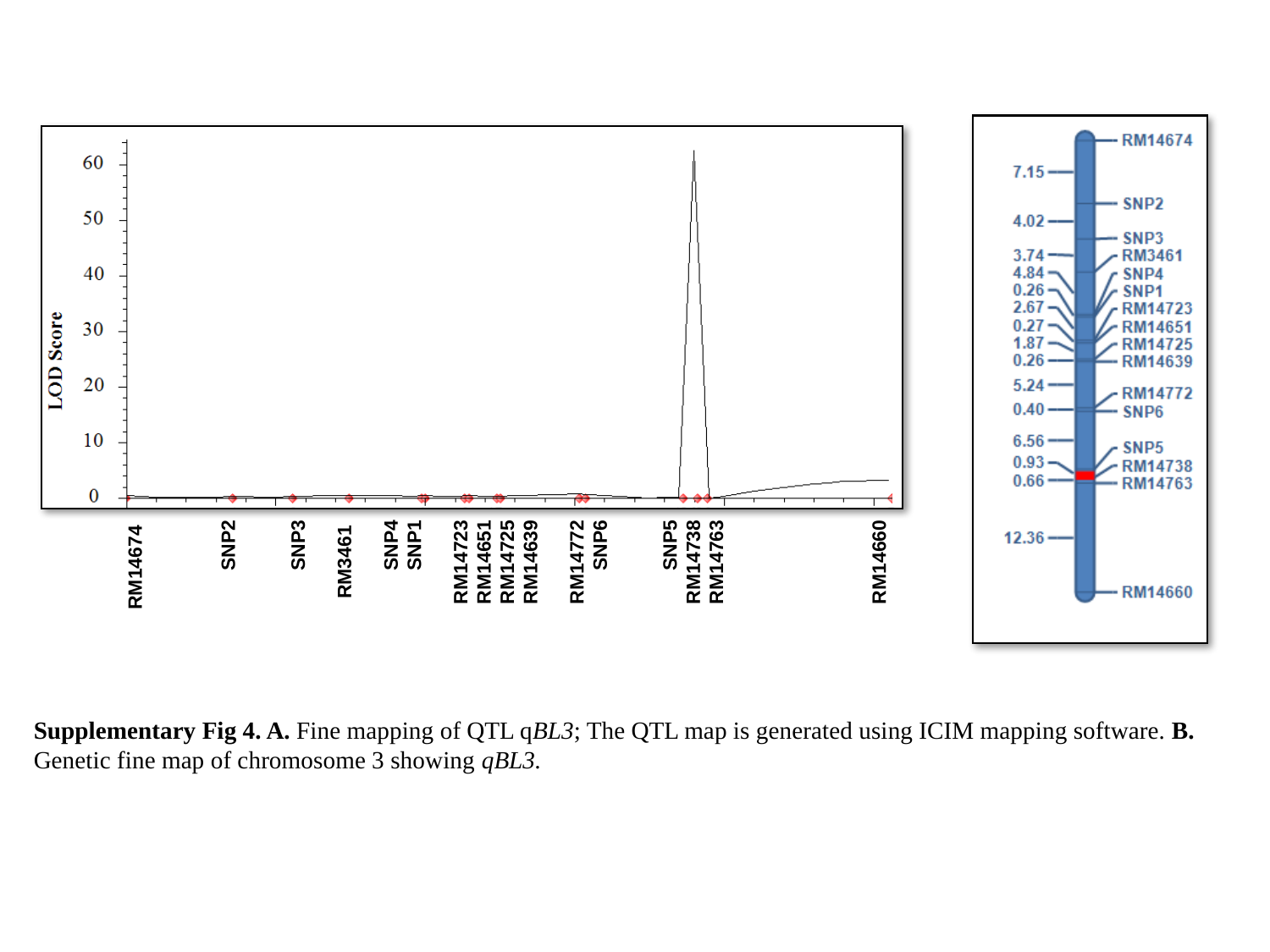

RM14674
SNP2
SNP3
RM3461
SNP4
SNP1
RM14723
RM14651
RM14725
RM14639
RM14772
SNP6
SNP5
RM14738
RM14763
RM14660
Supplementary Fig 4. A. Fine mapping of QTL qBL3; The QTL map is generated using ICIM mapping software. B. Genetic fine map of chromosome 3 showing qBL3.

## Slide 7
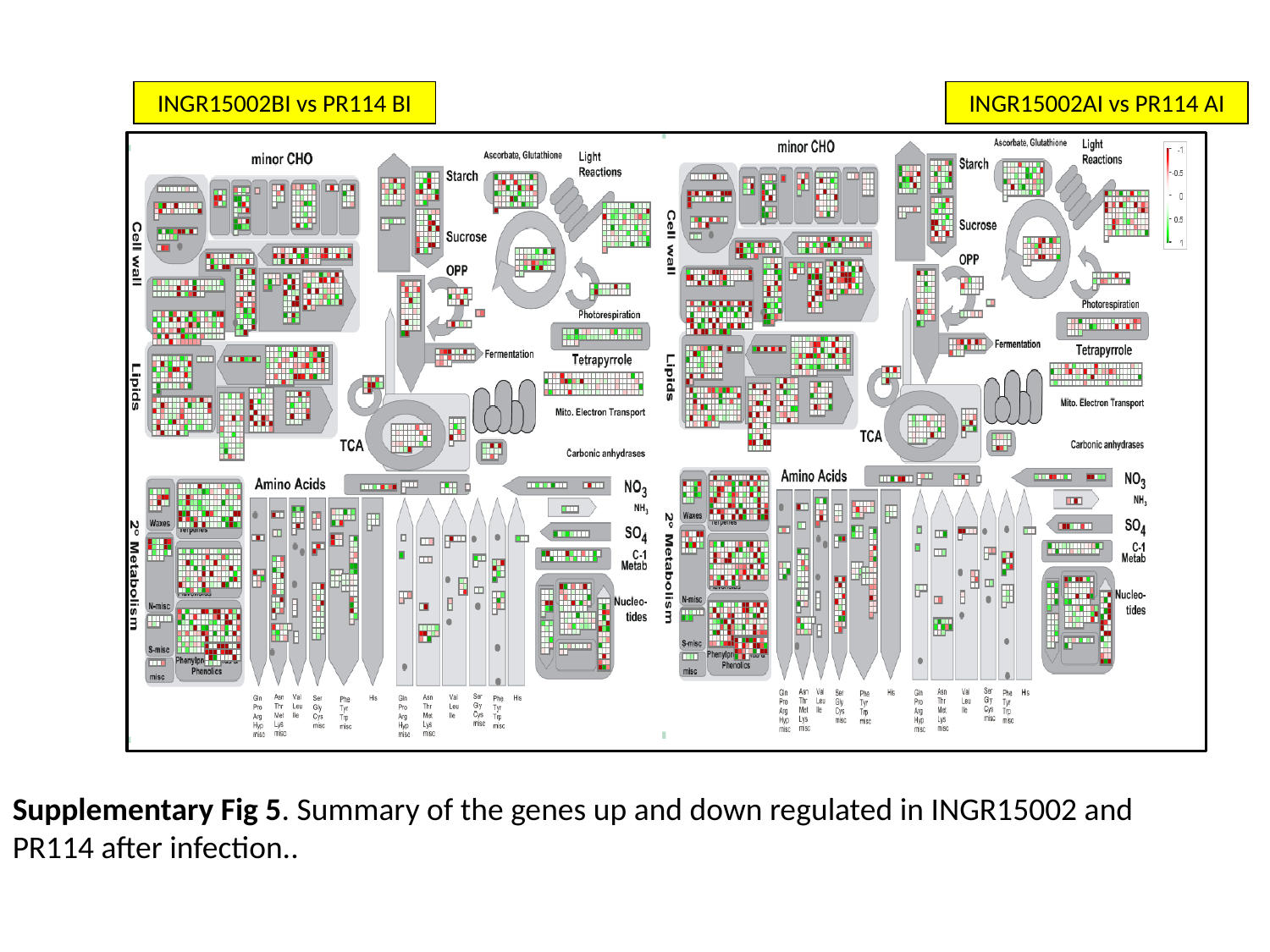

INGR15002BI vs PR114 BI
INGR15002AI vs PR114 AI
Supplementary Fig 5. Summary of the genes up and down regulated in INGR15002 and PR114 after infection..

## Slide 8
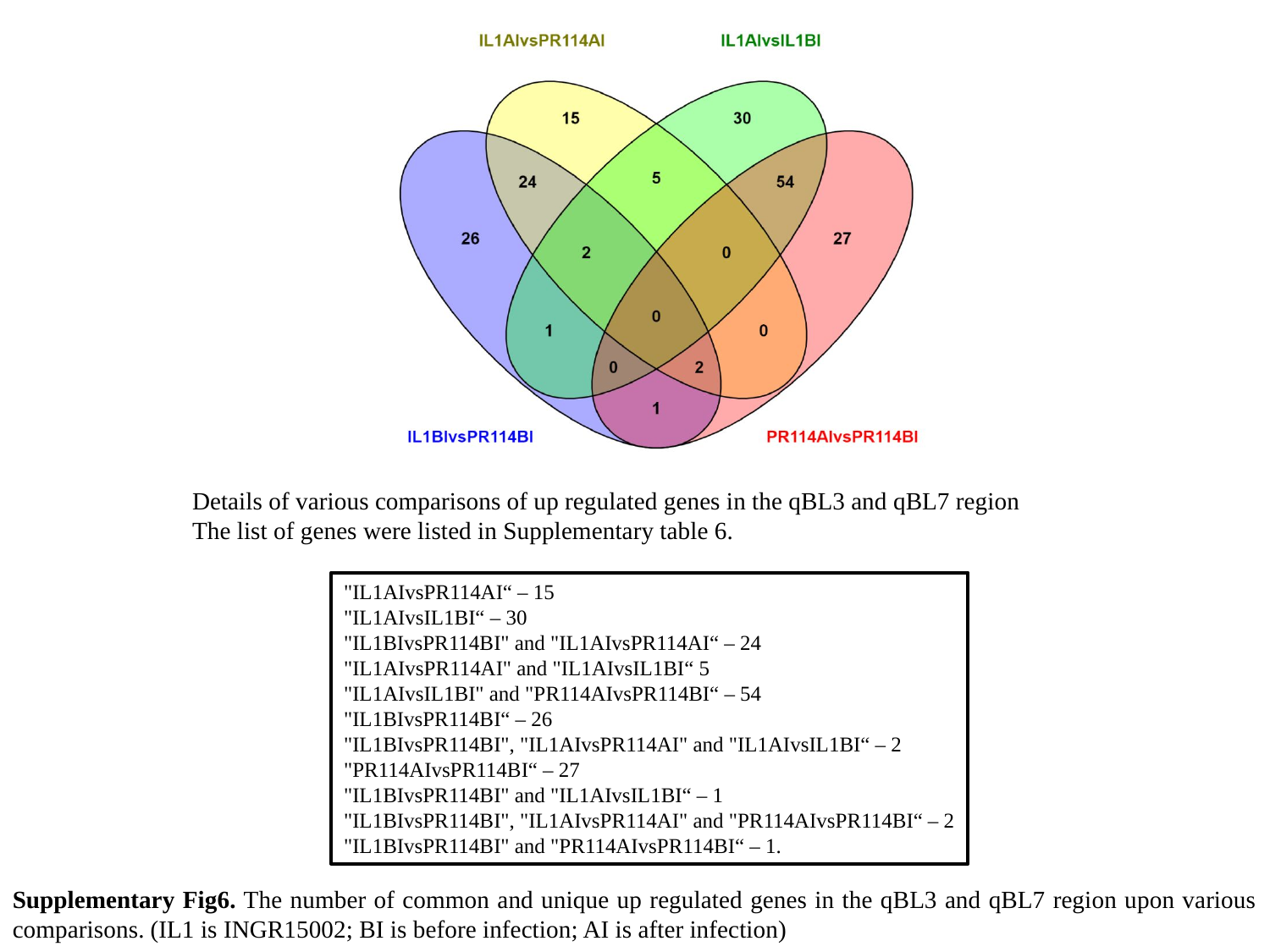

Details of various comparisons of up regulated genes in the qBL3 and qBL7 region
The list of genes were listed in Supplementary table 6.
"IL1AIvsPR114AI“ – 15
"IL1AIvsIL1BI“ – 30
"IL1BIvsPR114BI" and "IL1AIvsPR114AI“ – 24
"IL1AIvsPR114AI" and "IL1AIvsIL1BI“ 5
"IL1AIvsIL1BI" and "PR114AIvsPR114BI“ – 54
"IL1BIvsPR114BI“ – 26
"IL1BIvsPR114BI", "IL1AIvsPR114AI" and "IL1AIvsIL1BI“ – 2
"PR114AIvsPR114BI“ – 27
"IL1BIvsPR114BI" and "IL1AIvsIL1BI“ – 1
"IL1BIvsPR114BI", "IL1AIvsPR114AI" and "PR114AIvsPR114BI“ – 2
"IL1BIvsPR114BI" and "PR114AIvsPR114BI“ – 1.
Supplementary Fig6. The number of common and unique up regulated genes in the qBL3 and qBL7 region upon various comparisons. (IL1 is INGR15002; BI is before infection; AI is after infection)

## Slide 9
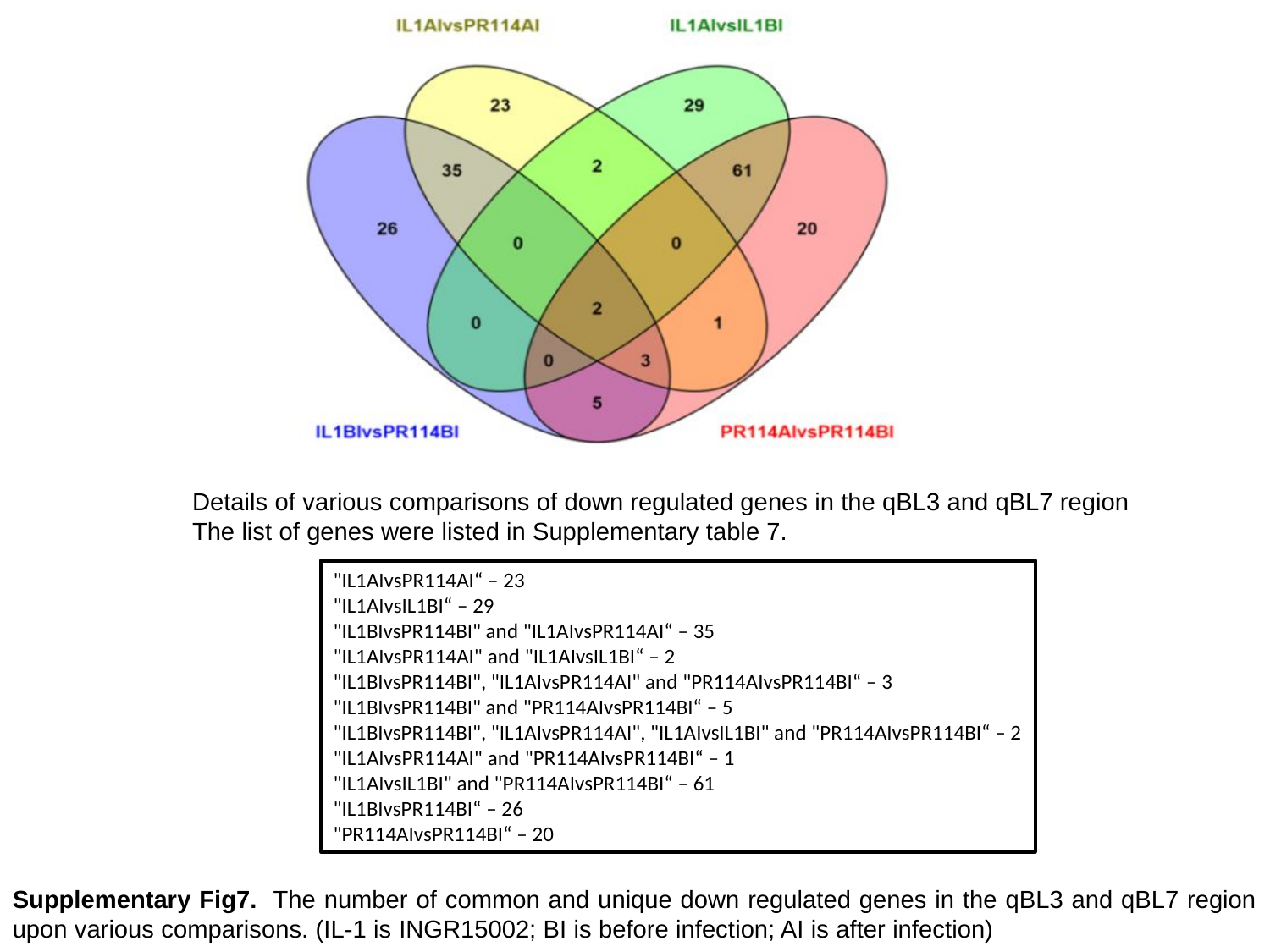

Details of various comparisons of down regulated genes in the qBL3 and qBL7 region
The list of genes were listed in Supplementary table 7.
"IL1AIvsPR114AI“ – 23
"IL1AIvsIL1BI“ – 29
"IL1BIvsPR114BI" and "IL1AIvsPR114AI“ – 35
"IL1AIvsPR114AI" and "IL1AIvsIL1BI“ – 2
"IL1BIvsPR114BI", "IL1AIvsPR114AI" and "PR114AIvsPR114BI“ – 3
"IL1BIvsPR114BI" and "PR114AIvsPR114BI“ – 5
"IL1BIvsPR114BI", "IL1AIvsPR114AI", "IL1AIvsIL1BI" and "PR114AIvsPR114BI“ – 2
"IL1AIvsPR114AI" and "PR114AIvsPR114BI“ – 1
"IL1AIvsIL1BI" and "PR114AIvsPR114BI“ – 61
"IL1BIvsPR114BI“ – 26
"PR114AIvsPR114BI“ – 20
Supplementary Fig7. The number of common and unique down regulated genes in the qBL3 and qBL7 region upon various comparisons. (IL-1 is INGR15002; BI is before infection; AI is after infection)

## Slide 10
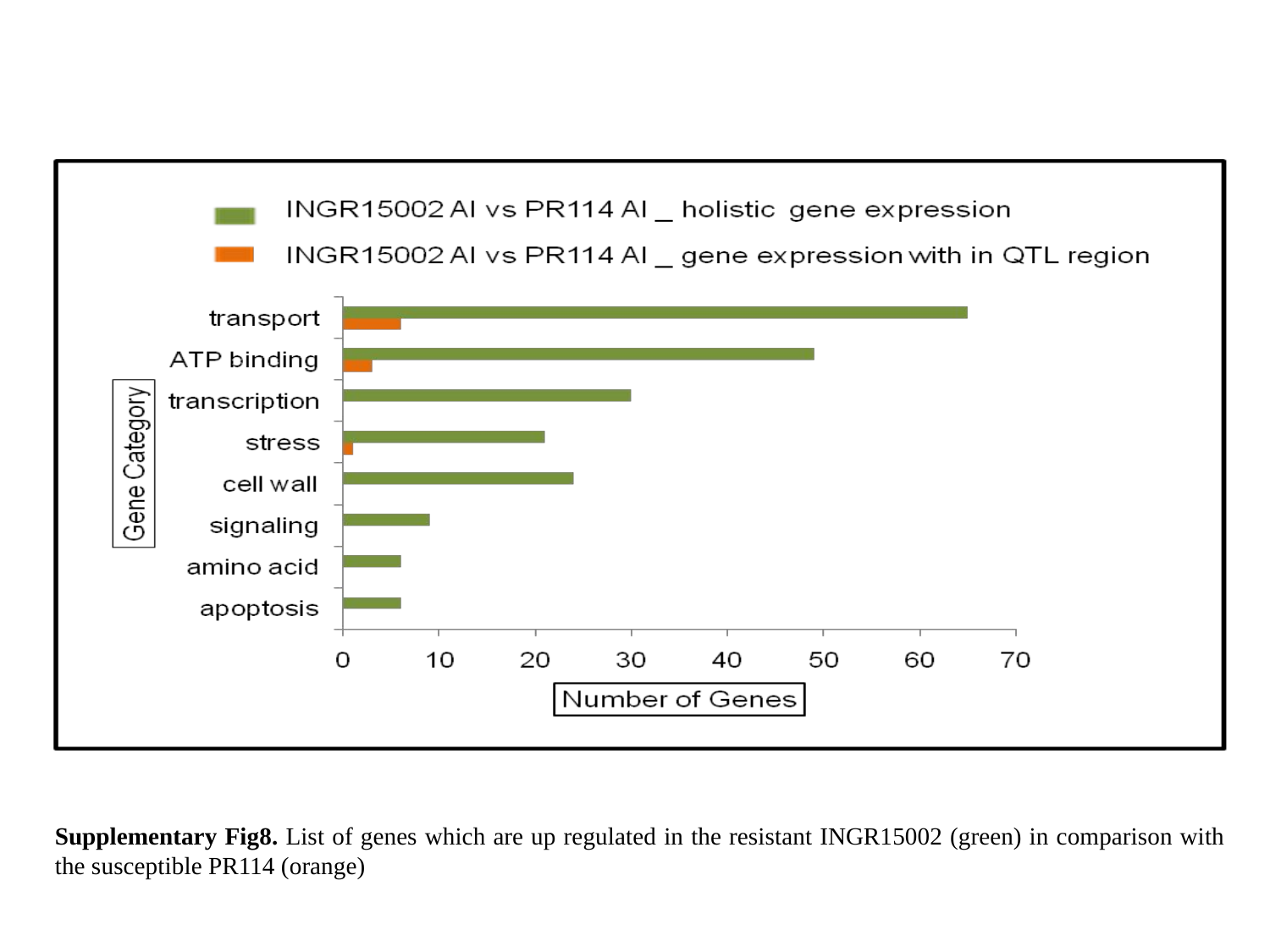

Supplementary Fig8. List of genes which are up regulated in the resistant INGR15002 (green) in comparison with the susceptible PR114 (orange)

## Slide 11
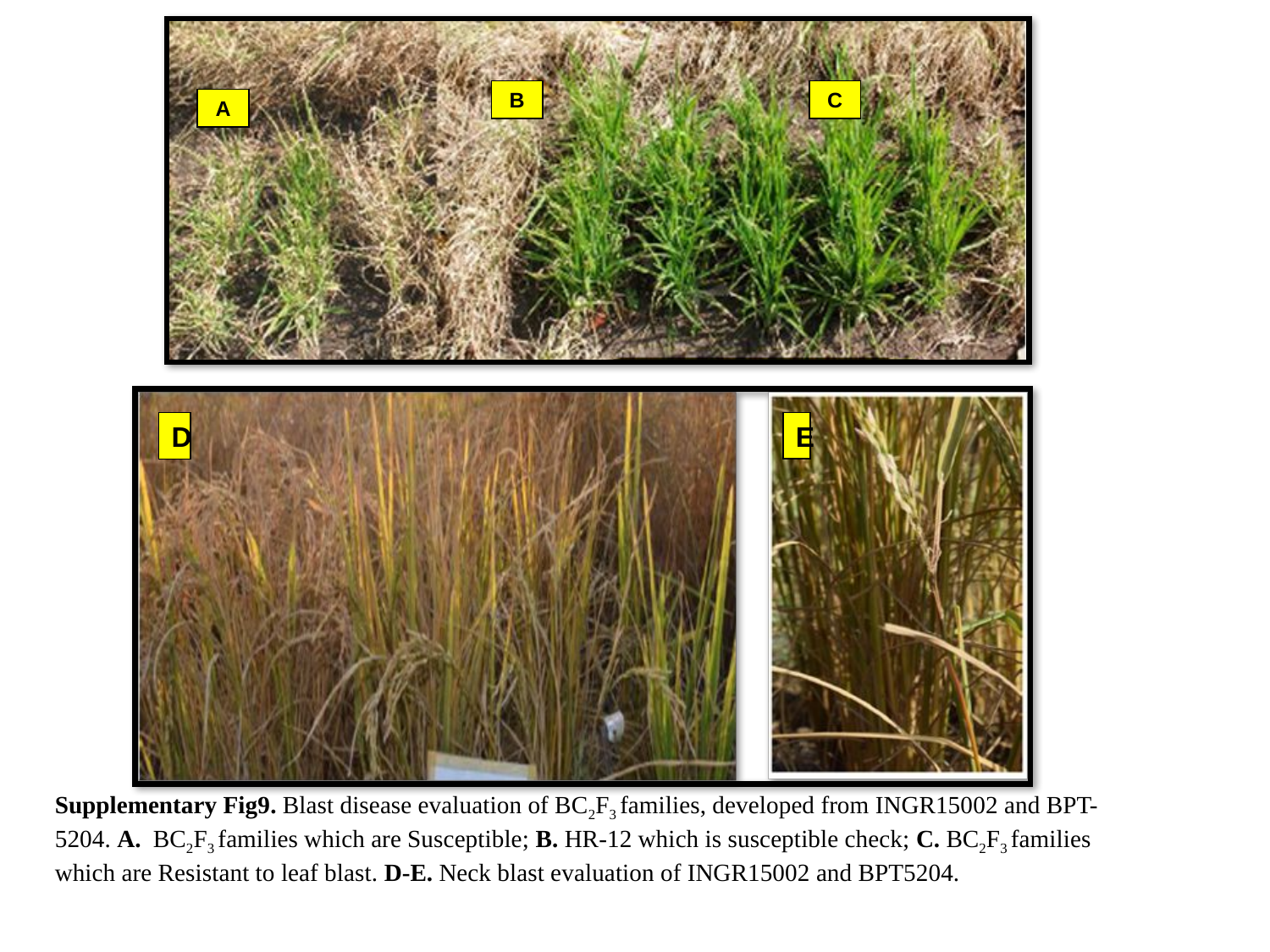

B
C
A
D
E
Supplementary Fig9. Blast disease evaluation of BC2F3 families, developed from INGR15002 and BPT-5204. A. BC2F3 families which are Susceptible; B. HR-12 which is susceptible check; C. BC2F3 families which are Resistant to leaf blast. D-E. Neck blast evaluation of INGR15002 and BPT5204.

## Slide 12
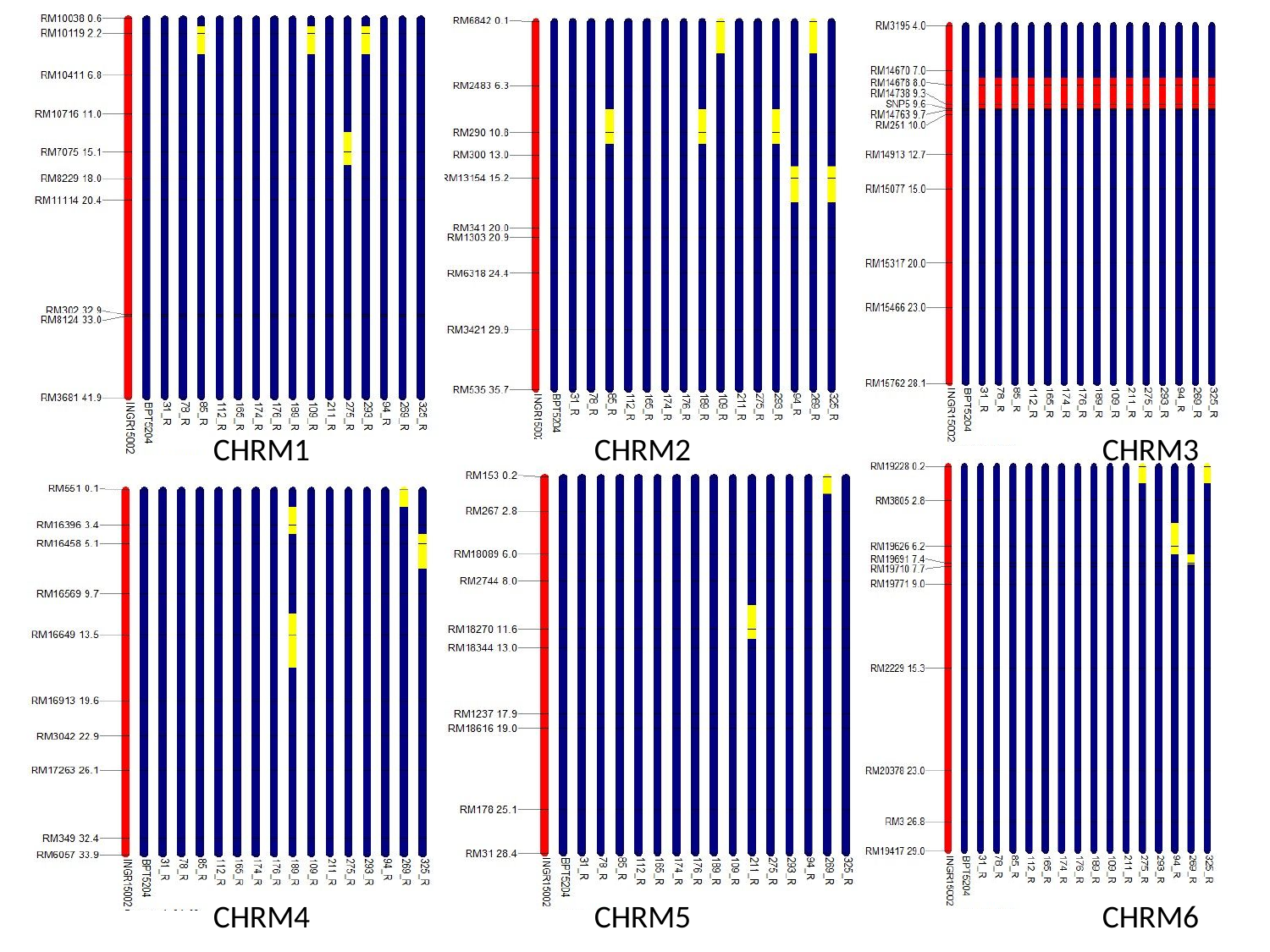

CHRM1			CHRM2				CHRM3
CHRM4			CHRM5				CHRM6

## Slide 13
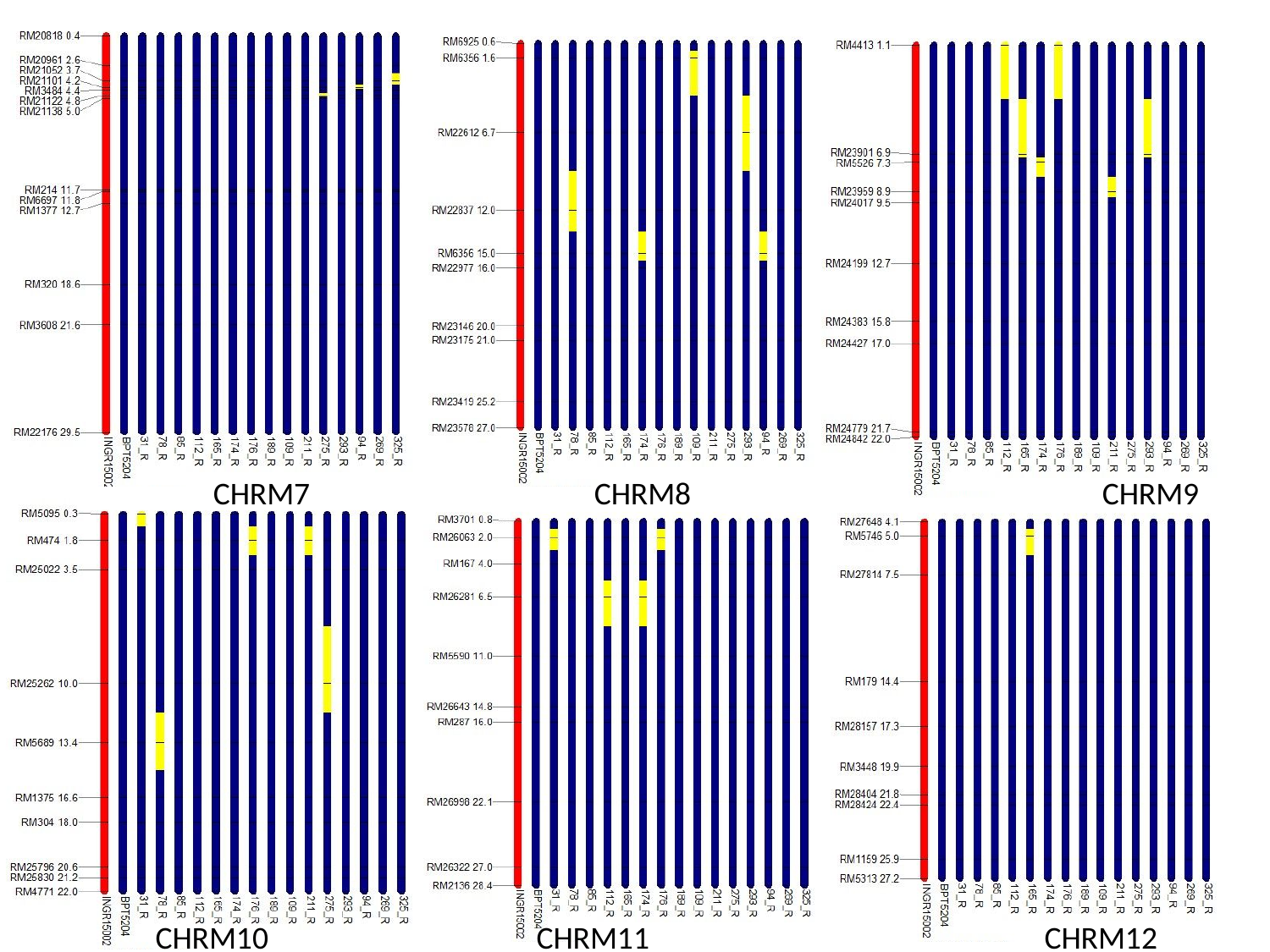

CHRM7			CHRM8				CHRM9
CHRM10			CHRM11				CHRM12

## Slide 14
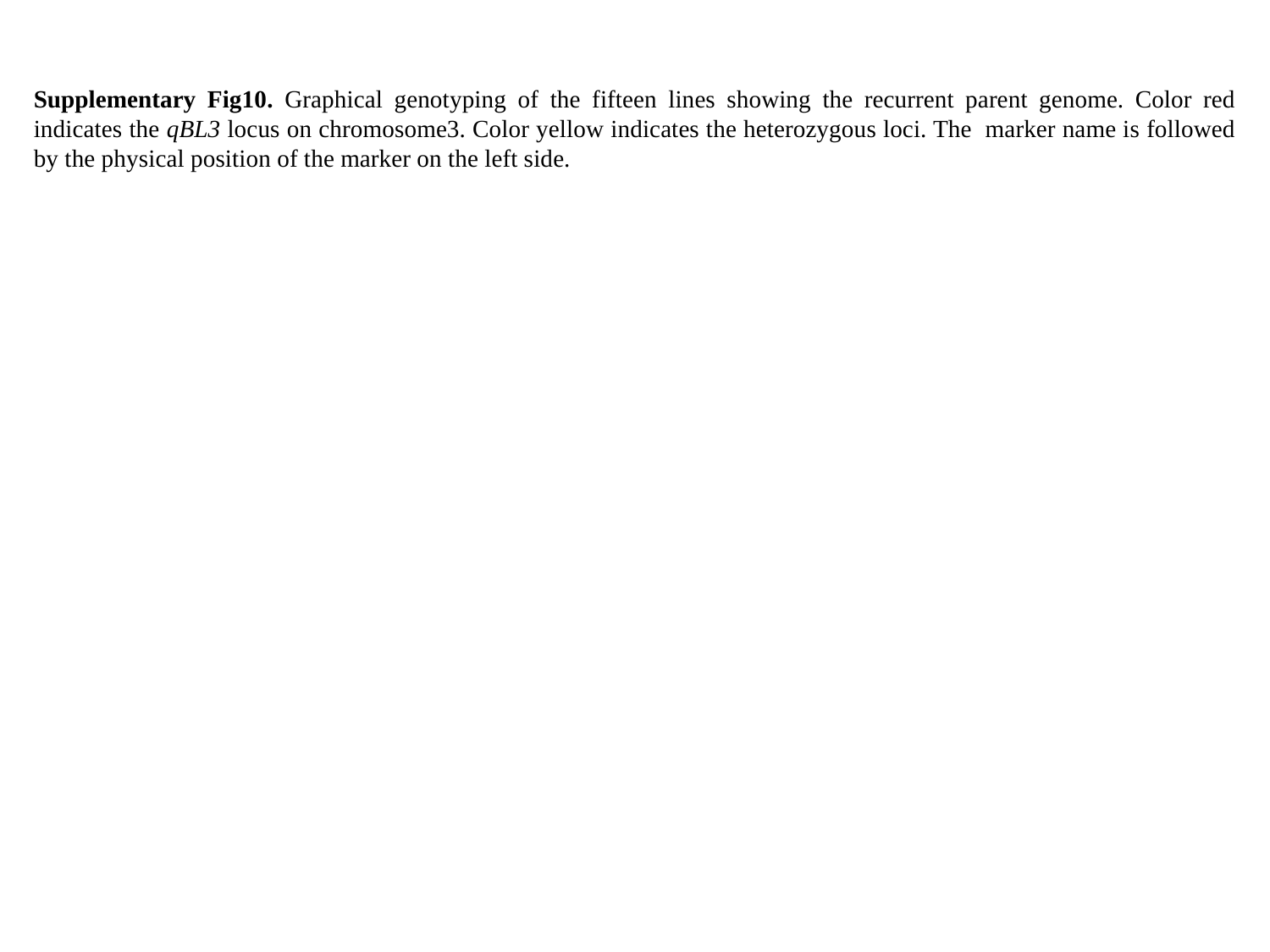

Supplementary Fig10. Graphical genotyping of the fifteen lines showing the recurrent parent genome. Color red indicates the qBL3 locus on chromosome3. Color yellow indicates the heterozygous loci. The marker name is followed by the physical position of the marker on the left side.
